# Hepatitis B virus P protein initiates glycolytic bypass in HBV-related hepatocellular carcinoma via a FOXO3/miRNA-30b-5p/MINPP1 axis

**DOI:** 10.1186/s13046-020-01803-8

**Published:** 2021-01-04

**Authors:** Wenbiao Chen, Jingjing Jiang, Lan Gong, Zheyue Shu, Dairong Xiang, Xujun Zhang, Kefan Bi, Hongyan Diao

**Affiliations:** 1 State Key Laboratory for Diagnosis & Treatment of Infectious Diseases, National Clinical Research Center for Infectious Disease, Collaborative Innovation Center for Diagnosis & Treatment of Infectious Diseases, The First Affiliated Hospital, College of Medicine, Zhejiang University, Hangzhou, 310003 China; 2 Microbiome Research Centre, St George and Sutherland Clinical School, University of New South Wales, Sydney, NSW Australia; 3 Department of Surgery, First Affiliated Hospital, Division of Hepatobiliary & Pancreatic Surgery, Zhejiang University School of Medicine, Hangzhou, 310000 China; 4 Key Lab of Combined Multi-Organ Transplantation, Ministry of Public Health, Hangzhou, 310000 China

**Keywords:** HBV, HCC, HBp, miRNA, MINPP1, Glycolysis

## Abstract

**Background:**

Hepatitis B virus (HBV) infection is a crucial risk factor for hepatocellular carcinoma (HCC). However, its underlying mechanism remains understudied.

**Methods:**

Microarray analysis was conducted to compare the genes and miRNAs in liver tissue from HBV-positive and HBV-negative HCC patients. Biological functions of these biomarkers in HBV-related HCC were validated via in vitro and in vivo experiments. Furthermore, we investigated the effect of HBV on the proliferation and migration of tumor cells in HBV-positive HCC tissue. Bioinformatics analysis was then performed to validate the clinical value of the biomarkers in a large HCC cohort.

**Results:**

We found that a gene, MINPP1 from the glycolytic bypass metabolic pathway, has an important biological function in the development of HBV-positive HCC. MINPP1 is down-regulated in HBV-positive HCC and could inhibit the proliferation and migration of the tumor cells. Meanwhile, miRNA-30b-5p was found to be a stimulator for the proliferation of tumor cell through glycolytic bypass in HBV-positive HCC. More importantly, miRNA-30b-5p could significantly downregulate MINPP1 expression. Metabolic experiments showed that the miRNA-30b-5p/MINPP1 axis is able to accelerate the conversion of glucose to lactate and 2,3-bisphosphoglycerate (2,3-BPG). In the HBV-negative HCC cells, miRNA-30b-5p/MINPP1 could not regulate the glycolytic bypass to promote the tumorigenesis. However, once HBV was introduced into these cells, miRNA-30b-5p/MINPP1 significantly enhanced the proliferation, migration of tumor cells, and promoted the glycolytic bypass. We further revealed that HBV infection promoted the expression of miRNA-30b-5p through the interaction of HBV protein P (HBp) with FOXO3. Bioinformatics analysis on a large cohort dataset showed that high expression of MINPP1 was associated with favorable survival of HBV-positive HCC patients, which could lead to a slower progress of this disease.

**Conclusion:**

Our study found that the HBp/FOXO3/miRNA-30b-5p/MINPP1 axis contributes to the development of HBV-positive HCC cells through the glycolytic bypass. We also presented miRNA-30b-5p/MINPP1 as a novel biomarker for HBV-positive HCC early diagnosis and a potential pharmaceutical target for antitumor therapy.

**Supplementary Information:**

The online version contains supplementary material available at 10.1186/s13046-020-01803-8.

## Background

Hepatocellular carcinoma (HCC) is one of the most prevalent malignant tumors globally, specifically in the Asian countries where hepatitis B virus (HBV) infection accounts for 90% cases of HCC [1]. This renders HBV infection as a major risk factor for HCC [2]. Although remarkable progress has been made to improve the early diagnostic and therapeutic approaches of HBV-related HCC, the detailed molecular mechanism on how HBV contributes to the HCC development is largely unclear. As a consequence, the breakthrough in clinically diagnostic and therapeutic strategies of HBV-related HCC still faces significant challenges.

The HBV genome is about 3.2 KB long and contains four open reading frames (ORFS), namely S gene, C gene, P gene, and X genome corresponding to encoding outer membrane protein (HBsAg, HBs), nucleocapsid protein (HBeAg/HBcAg, HBc), DNA polymerase (HBp), and HBxAg (HBx) [3]. The HBV genome is unequivocally required for viral replication [4]. HBx is essential to initiate and maintain transcription from cccDNA [5]. HBp, which is encapsulated in Dane particles during HBV replication, can repair short and missing strands in viral genomes to form complete double-stranded DNA [6]. In addition, these encoding proteins from HBV genome have been implicated in HBV-positive HCC development [3]. Several reports revealed that these proteins influence carcinogenic processes such as transcriptional activation, epigenetic regulation, and cell cycle progression in promoting the initiation of HBV-positive HCC [7]. Notably, emerging lines of evidence have shown that HBV proteins regulate non-coding RNAs, particularly microRNAs (miRNAs) that are involved in the replication of HBV [8].

The tumor development is closely related to metabolic reprogramming. Under low oxygen concentrations, cancer cells shifts to glycolysis from oxidation in converting glucose to lactate for the production of more energy for tumor growth, a rare phenomenon known as the Warburg effect [9]. Of note, gluconeogenesis is the fundamental characteristic of the liver. The transition from oxidation to glycolysis frequently observed in cancer cells is linked to tumorigenesis [10]. It was found that the aberrant expressions of anti-oncogene and oncogene involved in the metabolic pathways regulate the metabolic reprogramming in carcinogenesis [11]. Among the non-coding RNAs, it has been shown previously that miRNA regulates the metabolic reprogramming of cancer cells. Generally, miRNA regulates the expression of metabolism-related genes by inducing degradation of the target messenger RNA (mRNA) or inhibiting the gene expression at post-translational level [12]. Although previous studies have reported the relationship of miRNA with cancer metabolic regulatory network, the underlying mechanisms on how miRNA regulates the expression of metabolic pathway-related genes or which miRNA modulates tumor glycolysis remain largely unknown.

As a key component of glycolysis/gluconeogenesis pathway, the glycolytic bypass can transform 1,3-bisphosphoglycerate (1,3-BPG) to 2,3-bisphosphoglycerate (2,3-BPG), followed by producing 3-phosphoglycerate (3-PG) and 2-phosphoglycerate (2-PG) under the action of phosphatase (Figure S1, right) [13]. Additionally, the glycolytic bypass is a crucial pathway that regulates the oxygen transport function of hemoglobin. In anoxic conditions, such as altitude sickness, or acute respiratory distress syndrome (ARDS), the body initiates this pathway to provide more energy [14]. For a long time, researchers were confident that only a single enzyme, 2,3-BPG synthase/2-phosphatase (BPGM) was responsible for the glycolytic bypass reaction [14, 15] (Figure S1 right α). However, Jaiesoon et al recently revealed a new glycolytic reaction, which could bypass the formation of 3-PG and produce 2-PG (Figure S1, right β). They found that this reaction was catalyzed by multiple inositol-polyphosphate phosphatase 1 (MINPP1) [13]. Nevertheless, regardless of the conventional BPGM-mediated bypass or the new MINPP1-mediated bypass, the glycolytic bypass is a protective pathway confined in the red blood cells to correct hypoxia [16]. There is no existing evidence that the glycolytic bypass including the MINPP1-dependent reaction participates in metabolic reprogramming of tumors.

Herein, we first showed that HBV-positive HCC converts glucose to lactate geared towards providing energy for the proliferation of cancer cells through the glycolytic bypass. MINPP1, an anti-oncogene involved in the glycolytic bypass, was suppressed by an upstream miRNA (miRNA-30b-5p), thereby, facilitating the glycolytic bypass to produce more energy only in HBV-positive HCC. This is because HBp protein promotes the expression of miRNA-30b-5p through interaction with a transcription factor Forkhead Box O3 (FOXO3) to initiate the glycolytic bypass. Our study uncovered a novel mechanism, the HBp/FOXO3/miRNA-30b-5p/MINPP1 axis, contributing to the development of HBV-positive HCC through the glycolytic bypass, and suggested miRNA-30b-5p/MINPP1 as a potential target for treatment strategy against HBV-related HCC.

## Materials and methods

### Tissue specimens and cell lines

A total of 20 HBV-positive and 20 HBV-negative HCC liver tissue were collected from HCC patients who were firstly diagnosed and underwent surgical resection at the First Affiliated Hospital of Zhejiang University in 2018. The study focused on HCC patients without any prior treatment, and other viral infections including viral hepatitis caused by virus other than HBV (such as HAV, HCV, HEV) were excluded from the study. The human liver cell lines, including the HBV-positive Hep3B and HBV-negative Huh7, were obtained from the State Key Laboratory for Diagnosis and Treatment of Infectious Diseases, The First Affiliated Hospital, Zhejiang University. These cells were cultured in Dulbecco’s Modified Eagle’s Medium (DMEM) supplemented with 10% fetal bovine serum (Gibco™, New Zealand) at the 37 °C, humidified atmosphere with 5% CO_2_.

### Microarray and computational analysis

In total, 7 HBV-positive HCC liver tissue samples with HBsAg (+), HBeAg (+), HBcAb (+), and HBV-DNA > 10^4^ IU/ml and 7 HBV-negative HCC liver tissue samples were used for microarray sequence analysis by KangChen Biotech (Shanghai, China). The clinical characteristics of participants were presented in Table S1. Total RNA was isolated from each tissue specimen using TRIzol reagent (Invitrogen, Carlsbad, CA, USA) following the manufacturer’s instructions. In addition, sample labeling and array hybridization were performed as per the manufacturer’s standard protocols for Agilent One-Color Microarray-Based Gene Expression Analysis and miRNA Microarray System with miRNA Complete Labeling and Hyb Kit (Agilent Technologies, Palo Alto CA, USA). After the removal of rRNA (mRNA-ONLY™ Eukaryotic mRNA Isolation Kit, Epicentre), mRNA was purified from the total RNA. Each sample was then amplified and transcribed into fluorescent cRNA along the entire length of the transcripts without 3′ bias utilizing a random priming method (Arraystar Flash RNA Labeling Kit, Arraystar). The labeled cRNAs were purified by RNeasy Mini Kit (Qiagen, USA). The concentration and specific activity of the labeled cRNAs (pmol Cy3/μg cRNA) were measured by NanoDrop ND-1000 Spectrophotometer (Thermo Fisher, USA). The total miRNA from each sample was labeled with Cyanine 3-pCp under the action of T4 RNA ligase. After hybridization, washing, and fixation, 100 μl of hybridization solution was dispensed into the gasket slide to be assembled to the gene and miRNA expression microarray slide, which is scanned with Agilent DNA Microarray Scanner (part number G2505C). The array images were obtained and analyzed using Agilent Feature Extraction Software v10.7. The low intensity of mRNA and miRNA were discarded followed by normalization of signal intensities through GeneSpring GX v11.5.1 (Agilent Technologies, Palo Alto CA, USA). The differential expression patterns of mRNA and miRNA between HBV-positive and HBV-negative HCC samples were identified by volcano plot filtering. Thereafter, the significantly differential expression of mRNA and miRNA was verified by paired *t*-test, and fold change ≥2.0 with *P*-value ≤0.05 of mRNA and fold change ≥1.0 with *P*-value ≤0.05 of miRNA were being the threshold for statistical significance. Further, the significantly differential expression of mRNAs and miRNAs was clustered using the heatmap R package, and the biological function of the Kyoto Encyclopedia of Genes and Genomes (KEGG) in significantly differential mRNAs was analyzed via clusterProfiler R package. KEGG (http://www.kegg.jp/) is as an integrated database resource for biological interpretation of genomics, transcriptomics, proteomics, metagenomics, and other high-throughput data by pathway mapping [17]. Subsequently, the microarray data were uploaded to the Gene Expression Omnibus (GEO) database with an access number of GSE151441 for mRNA and GEO140400 for miRNA.

### Real-time quantitative polymerase chain reaction (RT-qPCR)

Total RNA from the tissue and cells was extracted using TRIzol reagent (Invitrogen, Carlsbad, CA) according to the manufacturer’s instruction. A total of 2 μg RNA of miRNA and 5 μg RNA of mRNA was reverse transcribed to cDNA using a miRNA cDNA Synthesis Kit with Poly (A) Polymerase Tailing (abm, Canada) and PrimeScript™ RT reagent Kit with gDNA Eraser (Takara, Dalian, China), respectively. The expression of miRNAs and mRNAs was analyzed by RT-qPCR system (Roche Diagnostics, Basel, Switzerland) using SYBR Premix Ex Taq™ (TaKaRa, Dalian, China). The primers for RT-qPCR are presented in Table S2. GAPDH/β-actin and U6 snRNA were used as the endogenous controls for mRNA and miRNA RT-qPCR, respectively. The 2^-△△Ct^ method was used to calculate the relative fold changes in the RNAs.

### Cell treatment and transfection

The cells were transfected using Lipofectamine 2000 (Invitrogen Corp., Carlsbad, CA, USA) following the manufacturer’s instruction. Small interfering RNA (siRNA) duplexes against MINPP1 gene was synthesized from GenePharma (Shanghai, China). The mimics, inhibitors, and corresponding negative control of miRNA-30b-5p were obtained from GENECHEM (Shanghai, China). Cells were transfected with 50 nM miRNA-30b-5p mimics in a six-well plate with 2 ml culture medium. The MINPP1 cDNA was cloned into PGLV3/H1/GFP lentiviral vectors using a lentivirus package (GenePharma, Shanghai, China). For lentivirus transfection, 5 × 10^6^ transducing units of lentivirus were transfected into cells. The sequence of miRNA-30b-5p mimics was sense, 5′-UGUAAACAUCCUACACUCAGCU-3′ and antisense, 5′-AGCUGAGUGUAGGAUGUUUACA-3′. Other sequences of siRNA and inhibitors used are shown in Table S2. Transfection of HBV DNA transient was performed by introducing 1.3mer HBV DNA (pHBV1.3) into Huh7 cells. The transfection efficiency of targets into the cells were tested by green fluorescence intensity from Green Fluorescent Protein (GFP) using fluorescence microscopy.

### Cell growth and function assays

The cellular proliferation capacity was assessed using the Cell Counting Kit-8 (CCK-8) (Beyotime Inst Biotech, China) when the cells were cultured in a hypoxic incubator (Heal Force, Shanghai, China) according to the manufacturer’s guideline. The absorbance of cells was measured with a microplate reader (Bio-Rad, Hercules, CA, USA) at a wavelength of 450 nm. The scrape motility assay was used to measure cell migration. Untreated cells were plated into culture inserts and then wound tip was created by scratched the cell monolayer with a sterile 200 μl pipette tip. Images of wound monolayers were captured using an inverted microscope (Olympus, Japan) at × 100 magnification at 0, 24, and 48 h post-wounding.

### Luciferase reporter assay

Luciferase reporters were constructed in the psiCHECK2 vector (Promega, Madison, WI, USA). The complete 3′ untranslated regions (UTR) of MINPP1 gene with the putative binding sites of miRNA-30b-5p were amplified and cloned into the psiCHECK2 vector to create psiCHECK2-MINPP1. Cells were then seeded in 24-well plates and allowed the cell density of 5000 cells per well overnight. The luciferase reporter was co-transfected with miRNA-30b-5p-wild-type mimics, miRNA-30b-5p mutant-type mimics, and control vectors into cells by Lipofectamine 2000. After 48 h of transfection, the luciferase activity was assessed by the dual-Luciferase Reporter Assay System (Promega, Madison, WI, USA).

### RNA fluorescence in situ hybridization (FISH)

Fresh HBV-positive and HBV-negative HCC liver samples were collected from surgical resection and immediately stored in liquid nitrogen, then the tissue were cut into 5-μm-thick sections and adhered to slides. The tissue was washed with phosphate-buffered saline (PBS) and fixed in 3.7% formaldehyde for 10 min. After washing the slides in 2 × sodium citrate buffer with a solution of 10% formamide, 4 μl fluorescent probes were added to the hybridization solution, which contained 10% dextran sulfate, 10% formamide, and 2 × sodium citrate buffer. Hybridization using MINPP1 and miRNA-30b-5p probes were performed overnight at 37 °C. The slides were rinsed twice for 20 min in 2× sodium citrate buffer with a solution of 10% formamide, then counterstained with 4′-6’diamidino-2-phenylindole (DAPI). The miRNA-30b-5p was labeled with 6-carboxy-fluorescein fluorophore (CY3) while MINPP1 was labeled with cyanine dye 3 (FAM). The location of MINPP1 and miRNA-30b-5p was detected using confocal laser scanning microscopy (Leica, Wetzlar, Germany).

### Measurement of metabolic indicators

Cells were cultured in phenol red-free DMEM for 15 h, then the medium was harvested for the measurement of metabolic indicators, including lactate production, glucose consumption, and 2-PG level. The glucose, lactate, and 2-BGP levels in the medium were quantified using glucose assay kit, lactate Assay kit, and 2-PG kit according to the manufacturer’s instructions (BioVision, Mountain View, USA).

### Xenograft in animal and histological immunohistochemistry

Six-week-old female BALB/c nude mice were purchased from the Beijing Experimental Animal Center (CSA, Beijing, China). For the establishment of xenograft tumors in animals, Hep3B cells stably expressing miRNA-30b-5p, inhibitors miRNA-30b-5p, MINPP1, and overexpressing MINPP1 were harvested and suspended in DMEM, and 3 × 10^6^ Hep3B cells in 200 μL of DMEM were subcutaneously injected into the proper bilateral flanks. Using the calipers, the tumor size was measured at an interval of every 2 days. After 4 weeks, the nude mice were sacrificed, their tumors dissected and the growth of subcutaneous tumors was measured. The tumors were then fixed with phosphate-buffered neutral formalin for histological examination. Notably, 44% PBS was used to buffer formaldehyde and extract the tissue, which were then embedded in paraffin and sectioned. The antibodies of MINPP1, miRNA-30b-5p, and proliferating cell nuclear antigen (PCNA) (Abcam, Cambridge, MA, USA) were subjected to immunohistochemical (IHC) analyses. The immunoreactivity was quantified by the horseradish peroxidase kit (BioGenex, Fremont, CA, USA). Moreover, the tissue were counterstained with hematoxylin-eosin (HE). All animal experiment procedures were performed according to the Animal Care Commission of Zhejiang University.

### Bioinformatics and statistical analysis

The research data, constituted of RNA sequencing (RNA-Seq), miRNASeq, and clinical follow-up information, were downloaded from The Cancer Genome Atlas (TCGA) database. Sixty HBV-positive HCC and 211 HBV-negative HCC samples were selected for subsequent analysis. In addition, the GSE55092 dataset was collected, including 49 HBV-positive HCC samples from the GEO database. Student’s *t*-test and Wilcoxon test were performed to assess the differences in variance between indicators for normally distributed and non-normally distributed data respectively. On the other hand, Kruskal–Wallis tests were used to perform statistical analysis for nonparametric testing of three or more datasets. The NetworkD3 R packages were used to depict the alluvial diagram of mRNA and corresponding KEGG pathways. A alluvial diagram is a kind of plane diagram that can visually show the changes between groups, time series, complex multi-attribute, and multi-correlation [18]. The mRNA-miRNA regulatory network was analyzed by Weighted Correlation Network Analysis (WGCNA) R package and was depicted using Cytoscape software. Additionally, miRanda (http://www.microrna.org/microrna/home.do) and TargetScan (http://www.targetscan.org/vert_72/) tools were used to predict the direct target of 3′ UTR in MINPP1. The association between MINPP1 and miRNA in the TCGA was calculated by the Pearson correlation coefficient, with a correlation diagram being used to depict the relationship by Corrplot R package. HBV-positive HCC samples of TCGA were divided into high and low expression groups by the median MINPP1 expression. The Kaplan–Meier (KM) method was used to depict the survival curve and estimate the survival probability of patients which was further examined by the log-rank test. The Forest plot was performed using the Forest plot R package to present the univariate Cox regression analysis for statistical summary of clinical indicators and MINPP1. The relationship between MINPP1 and genes from TCGA and GSE55092 datasets was analyzed using coexpression models i.e., WGCNA R package. Moreover, the association between miRNA from KEGG and predicted genes were revealed through the Hmisc R package. Of note, WGCNA is a systems biology method applied in constructing scale-free networks using gene expression data. The overlapped genes associated with MINPP1 between TCGA and GSE55092 were used for KEGG and gene ontology (GO) analyses by clusterProfiler R package and GOplot R package respectively. For statistical analyses, SPSS software and R package were used, and *P* < 0.05 was considered statistically significant.

## Results

### MINPP1 involved in the glycolytic bypass impairs the growth of HBV-positive HCC

**Fig. 1:**
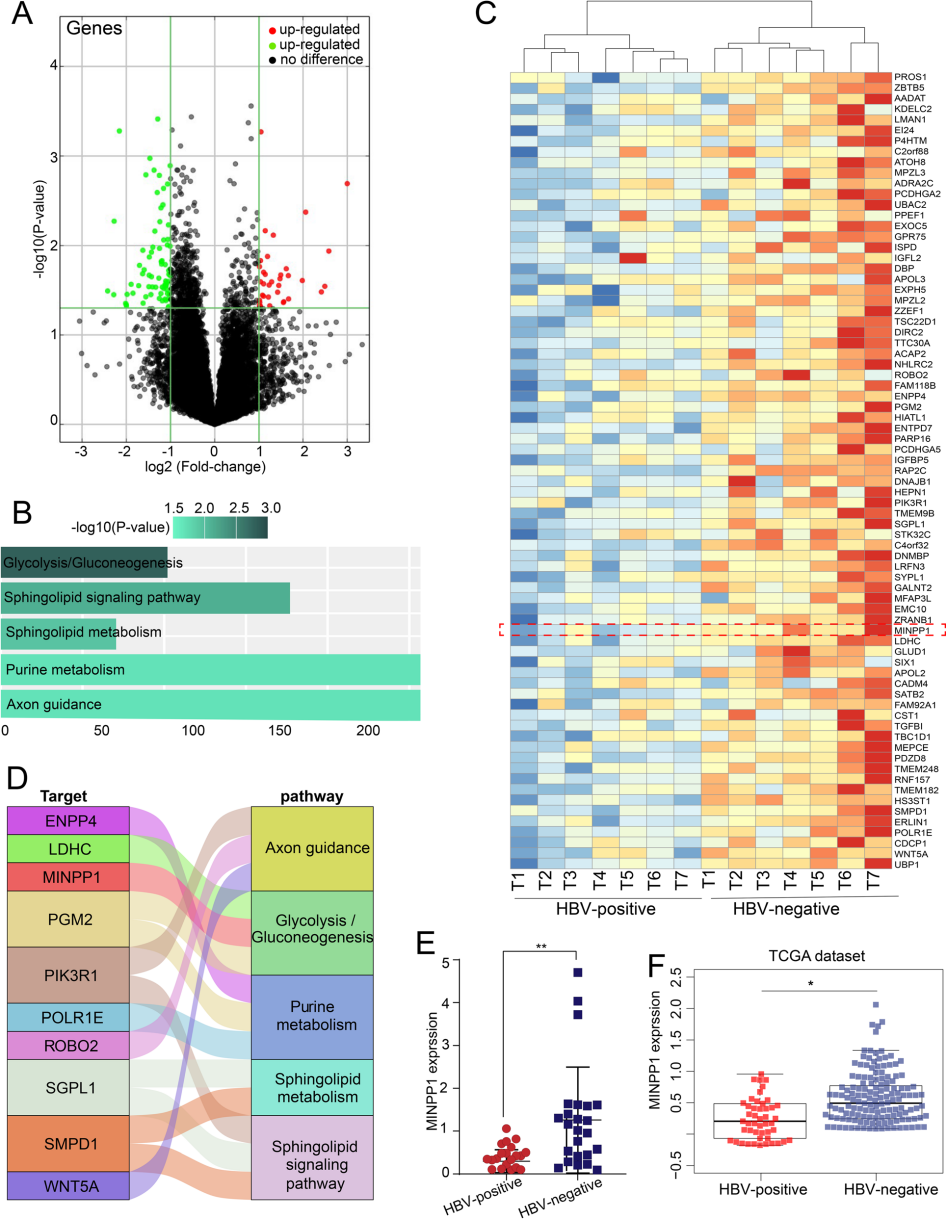
Identification of MINPP1 by microarray and bioinformatics analyses. **a** A Volcano Plot was used to identify differentially expressed genes between HBV-positive and HBV-negative HCC tissues. **b** KEGG analysis of the down-regulated genes in HBV-positive and HBV-negative HCC tissues. **c** Heatmap clustering of down-regulated genes in HBV-positive and HBV-negative HCC. **d** Alluvial diagram represents genes involved in biological pathways. **e** RT-qPCR results of MINPP1 expression in 20 paired HBV-positive and HBV-negative HCC tissues. **e** The expression level of MINPP1 was verified in a HCC cohort from the TCGA database. ^*^P < 0.05, ^**^P < 0.01

Since it has been shown that HBV infection is linked to increased risk of HCC, we first investigated the association of HBV infection with the development of HBV-related HCC. To address the role of HBV infection, we conducted microarray analysis in 7 pairs of HBV-positive and HBV-negative HCC samples. In total, 35 up-regulated and 75 down-regulated genes (fold change ≥2.0, *P* ≤ 0.05) exhibited significantly differential expression between HBV-positive and HBV-negative tissues (Fig. 1a; Table S3). Afterward, KEGG analysis was performed to investigate the biological function of the differentially expressed genes, the results showed that the down-regulated genes are involved in 3 metabolic pathways, including glycolysis/gluconeogenesis, purine metabolism, and sphingolipid metabolism (Fig. 1b). The heatmap clustering was applied to show that the 75 down-regulated genes exhibit significant differences in expression between HBV-positive and HBV-negative HCC tissue samples (Fig. 1c). The glycolysis/gluconeogenesis pathway related to tumorigenesis is involved in HCC progression [19, 20]. The alluvial diagram showed that LDHC, PGM2, and MINPP1 were enriched in glycolysis/gluconeogenesis (Figs. 1d, S1). Despite MINPP1 is involved in the glycolytic bypass in red blood cells [16], no evidence has supported a role of MINPP1 in regulating tumorigenesis via the glycolytic bypass (Figure S1, right β). In this study, we focused on whether MINPP1 could regulate the glycolytic bypass in HBV-positive HCC development. We hereby tested the expression of MINPP1 in 20 paired HBV-positive and HBV-negative HCC tissues. It was found that the expression of MINPP1 was significantly down-regulated in HBV-positive HCC compared to HBV-negative HCC (*P* < 0.01) (Fig. 1e). In addition, a similar result was obtained from the comparison between 60 HBV-positive and 211 HBV-negative HCC samples from the TCGA database (Fig. 1f), which also confirmed the MINPP1 was down-regulation in HBV-positive HCC patients.

**Fig. 2:**
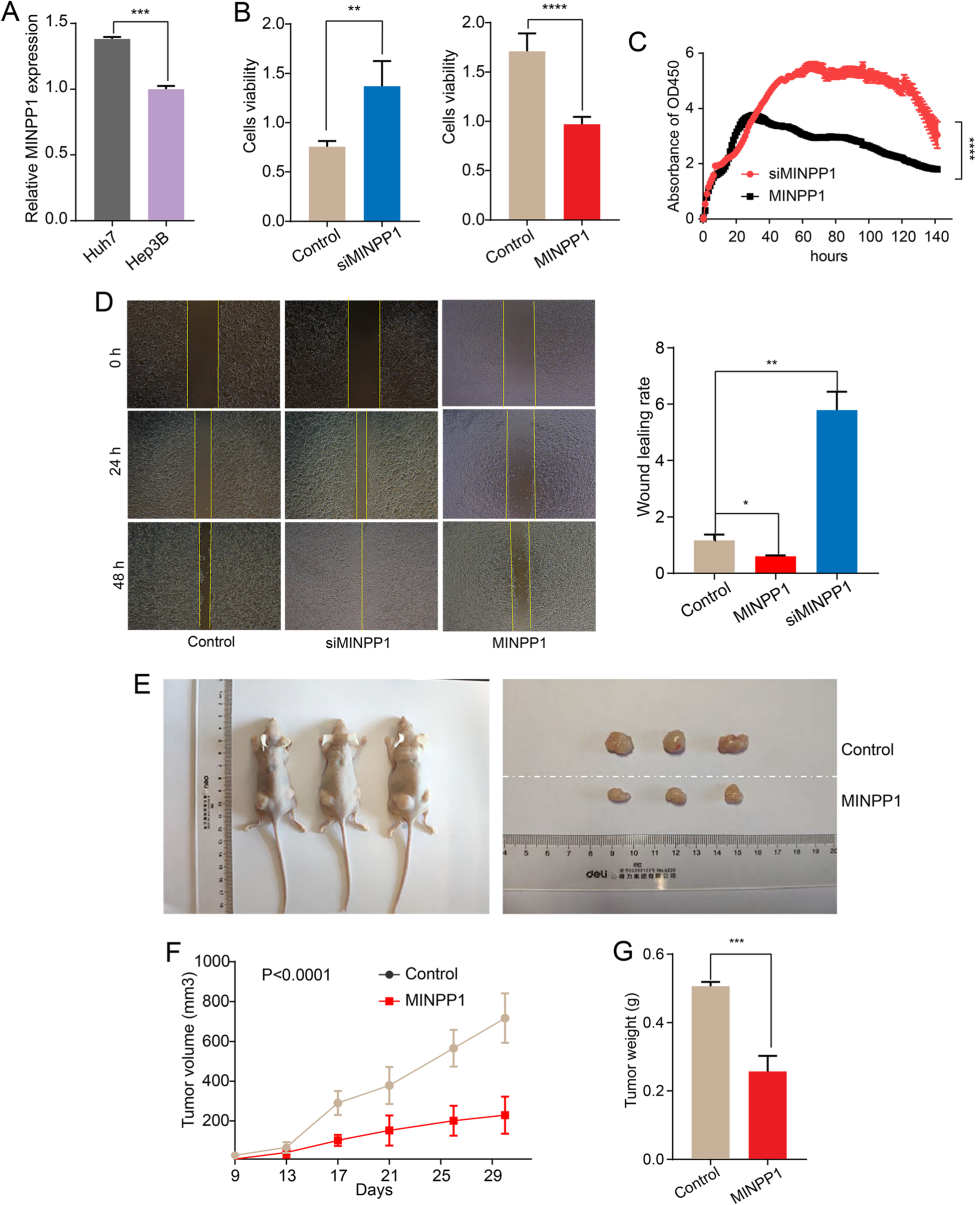
MINPP1 gene was identified to be an anti-oncogene in HBV-positive HCC. **a** RT-qPCR results showing MINPP1 expression level in hepatoma cell lines. **b** Cell proliferation was assessed with the CCK-8 assay in Hep3B cells with MINPP1 knockdown (left) and overexpression (right). **c** The real-time cell proliferation of Hep3B cells with MINPP1 knockdown and overexpression was determined by CCK-8 assay. **d** Scrape motility were measured by cell migration assays at 0, 24, and 48 h in the Hep3B cells with MINPP1 knockdown or overexpression. The wound healing rate was tested by Image J software. **e** Left: The tumor formation in nude mice injected subcutaneously with Hep3B cells overexpressing MINPP1 (right) and control cells (left). Right: The harvested xenograft tumors. **f** The tumor volume was measured after nude mice were injected with Hep3B cells overexpressing MINPP1 and control cells. **g** The tumor weight was measured after the nude mice were injected with the Hep3B cells overexpressing MINPP1 and control cells. ^**^P < 0.01, ^***^P < 0.001, ^****^P < 0.0001

To further explore the biological function of the MINPP1 gene in HBV-positive HCC, we examined the expression level of MINPP1 in different hepatoma cell lines. The result showed that the HBV-positive HCC cell line Hep3B has lower MINPP1 expression than the HBV-negative HCC cell line Huh7 (Fig. 2a), which is in consistent with the results in HCC tissue (Fig. 1e, f). We constructed Hep3B cell lines that stably overexpress MINPP1 or were knocked down for MINPP1 (Figure S2A, B). Through CCK-8 assays, MINPP1 knockdown significantly increases the proliferative viability of Hep3B, whereas overexpressed MINPP1 yielded the opposite results (Fig. 2b). In consistent with this finding, real-time cell proliferation assay also showed that overexpressed MINPP1 resulted in a lower proliferative capacity in Hep3B cells compared to that of knocked down MINPP1 (Fig. 2c). Scrape motility analysis further showed that knockdown of MINPP1 increased the invasion ability of Hep3B cells, while overexpression of MINPP1 decreased the invasion ability (Fig. 2d). The reverse effect of MINPP1 expression on the proliferation and invasion of HBV-positive liver cell lines leaded to a hypothesis that MINPP1 may suppress tumor growth in HBV-positive HCC tissue. To test this hypothesis, we injected the Hep3B cells stably overexpressing MINPP1 and those expressing MINPP1 at normal level (control) into the left and right bilateral flanks of nude mice, respectively. It was witnessed that the growth, volume, and weight of tumor lumps in the xenograft mouse model were significantly decreased in mice injected with cells overexpressing MINPP1 as compared to the control groups (Fig. 2e-g). HE and PCNA analysis of tumor pathological tissue further revealed that overexpression of MINPP1 gene inhibits the proliferation of tumor cells (Figure S3). The above results suggested that MINPP1 acts as an anti-oncogene via the glycolytic bypass to suppress the HBV-positive HCC development.

### Validation of MINPP1 function by WGCNA analysis in the HBV-positive HCC cohorts

To further investigate the biological roles of MINPP1 in the HBV-positive HCC, the gene expression profiles related to MINPP1 were used to analyze the co-expression models from different databases by the WGCNA R package. For 49 HBV-positive HCC from the GSE55092 cohort, the WGCNA algorithm was performed using an unsigned topology overlap matrix, applying a soft threshold power of β = 5 to achieve an approximate scale-free topology. A total of 23 models (Figure S4A) were obtained based on 3 parameters including height = 0.25, deepSplit = 2, and minModuleSize = 30. The most significant model was brown with 459 genes. A similar method was adopted to analyze the 60 HBV-positive HCC from the TCGA cohort, as a result, 24 models (Figure S4B) were obtained according to height = 0.1, deepSplit = 2, and minModuleSize = 25 parameters. Again, the most significant model was brown with 365 genes. The overlapped 53 genes between these two cohorts were subjected to GO and KEGG analyses. GO analysis showed that these genes were associated with cell metabolism and cell cycle, such as regulation of ATP metabolic processes and mitotic G1 DNA damage checkpoint (Figure S4C). Specifically, the MINPP1 gene was involved in ATP metabolic process. KEGG analysis showed that these genes were connected with the metabolic and tumorigenesis-related process i.e., glucagon signaling and Wnt signaling pathways (Figure S4D). Notably, glycolysis/gluconeogenesis was also found in this enrichment. These results of bioinformatics analyses further indicated that MINPP1 is a potential biomarker to differentiate between HBV-positive and HBV-negative HCC tumorigenesis.

### Identification of miRNA-30b-5p as a target of MINPP1 gene in HBV-positive HCC

**Fig. 3:**
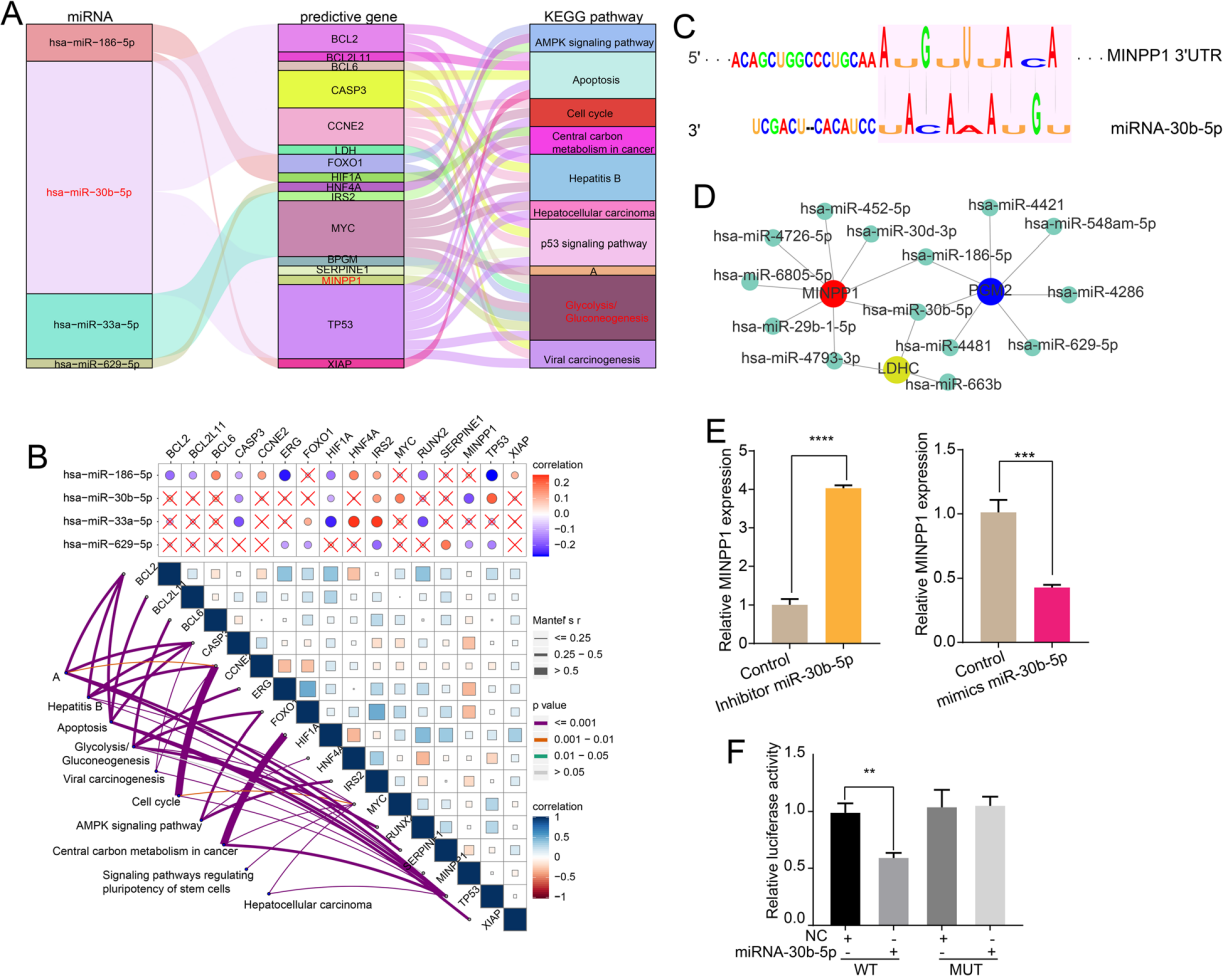
miRNA-30b-5p was identified to be a target of MINPP1 contributing to the development of HBV-positive HCC. **a** Alluvial diagram showing the association of 10 most significant pathways involved in HCC development with miRNAs and their predicted target genes. A: Signaling pathways regulating pluripotency of stem cells. **b** Associational of miRNAs and their predicted targeted genes with pathways. A: p53 signaling pathway. **c** Prediction of binding sites of miRNA-30b-5p on the MINPP1 transcript. **d** The interaction network of miRNA and mRNA. The red, blue, and yellow dot represent MINPP1, PGM2, and, LDHC, respectively, involved in glycolysis/gluconeogenesis pathway. The green dots represent differentially expressed miRNAs associated with three genes. **e** RT-qPCR results of MINPP1 expression level in Hep3B cells after treatment with inhibitor and mimics of miRNA-30b-5p. **f** Relative activity of luciferase reporters with MINPP1-UTR after co-transfection with miRNA-30b-5p mimics or mutant in Hep3B cells. ^**^P < 0.01, ^***^P < 0.001, ^****^P < 0.0001. ^**^P < 0.01, ^***^P < 0.001, ^****^P < 0.0001

The identification of differentially expressed miRNAs was performed on 7 pairs of HBV-positive and HBV-negative HCC tissue samples (same specimen on mRNA analysis) using microarray analysis, and the results showed that the expression of 24 miRNAs was significantly up-regulated in HBV-positive HCC compared to HBV-negative HCC (fold change ≥1.0, *P* ≤ 0.05) (Figure S5A, B; Table S4). To elucidate the biological function of these miRNAs involved in the pathogenesis of HBV-positive HCC, the target genes of 24 miRNA (score > 0.6) were predicted based on the interactions of human miRNAs and their target genes in RAID V3.0 database. Then the target genes were subjected to KEGG analysis, and 10 pathways that were most significant for HCC development, such as Hepatitis B and HCC were selected to depict the association as shown by alluvial diagram (Fig. 3a). It was observed that MINPP1 was predicted as the target gene of miRNA-30b-5p involved in the glycolysis/gluconeogenesis pathway. Notably, miRNA-30b-5p was the most important miRNA connected with these 10 HCC related pathways (Fig. 3a). Hence, this study allows to speculate that miRNA-30b-5p is a crucial target in the pathogenesis of HCC. miRNA-genes association analysis revealed that miRNA-30b-5p was negatively related to MINPP1, and MINPP1 was negatively associated with other predicted target genes (Fig. 3b), which suggested a potentially modulated relationship between MINPP1 and miRNA-30b-5p. Previous studies reported that genes could be regulated by upstream miRNA by complementing with the 3 ‘UTR region of the target genes [21]. We therefore searched TargetScan and miRanda databases and confirmed that miRNA-30b-5p indeed contains target sites for the 3 ‘UTR region of MINPP1 (Fig. 3c). In addition, the interaction network revealed that the 3 genes (MINPP1, PGM2, and LDHC) of glycolysis/gluconeogenesis pathway showed a mutual interaction with several miRNAs, and that MINPP1 connected with 8 miRNAs, including miRNA-30b-5p (Fig. 3d). Using RT-qPCR, the inverse correlation between MINPP1 and miRNA was verified in Hep3B cells treated with mimics or inhibitors of miRNA-30b-5p (Fig. 3e). Luciferase reporter assay showed that luciferase activity of MINPP1 was significantly inhibited in the wild type (WT) groups compared to the control group, and that this inhibition could be reversed through the mutation of the binding site (MUT) (Fig. 3f). In addition, HBV-positive HCC samples that detected the expression of both miRNA and MINPP1 were screened from the TCGA database, and 58 samples with 736 miRNAs were found. After calculation by Pearson correlation coefficient with *p* < 0.01 and |R| > 0.3, 9 miRNAs were obtained, which were negatively associated with MINPP1, including miRNA-30b-5p (Figure S6A). The interaction network also indicated MINPP1 was related to miRNA-30b-5p (Figure S6B). All the above results suggested that miRNA-30b-5p is a target of MINPP1, and that miRNA-30b-5p potentially regulates the expression of MINPP1.

### Up-regulated miRNA-30b-5p promotes the development of HBV-positive HCC and its interation with MINPP1

**Fig. 4:**
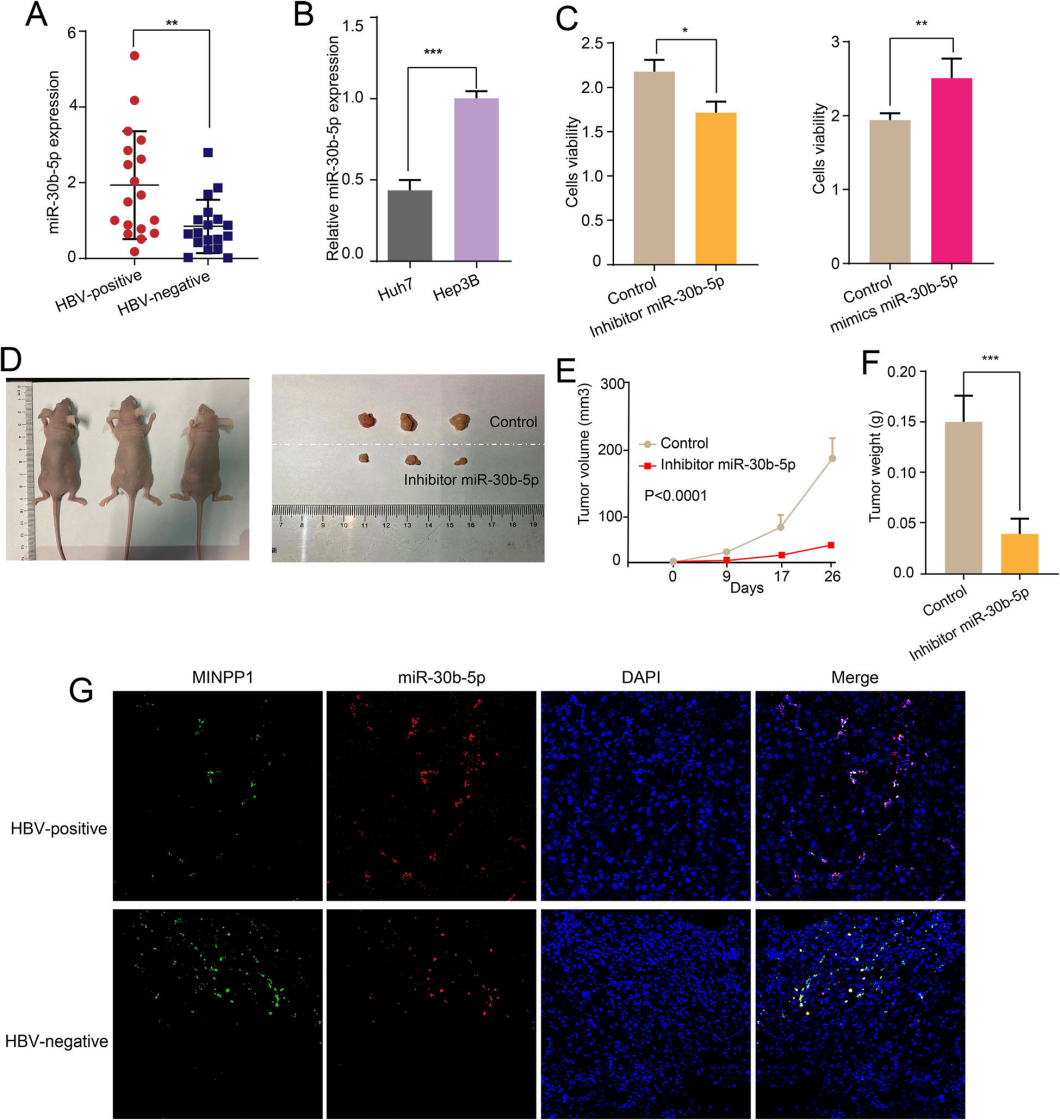
miRNA-30b-5p is an oncogene that regulates MINPP1. **a** RT-qPCR results of miRNA-30b-5p expression level in 20 paired HBV-positive and HBV-negative HCC tissues. **b** RT-qPCR results of miRNA-30b-5p expression level in hepatoma cell lines. **c** Cell proliferation was assessed with the CCK-8 assay in Hep3B cell in which miRNA-30b-5p was inhibited (left) and overexpressed (right). **d** Left: The tumor formation in nude mice injected subcutaneously with Hep3B cell in which miRNA-30b-5p was inhibited (right) and control (left). Right: The harvested xenograft tumors. **e** The tumor volume was measured after the nude mice were treated with Hep3B cell in which miRNA-30b-5p was inhibited and control. **f** Tumor weight was measured after the nude mice were treated with Hep3B cells transfected with the inhibitor of miRNA-30b-5p and control construct. **g** RNA FISH revealed the co-localization of miRNA-30b-5p and MINPP1 in the HBV-positive HCC sample. The miR-30b-5p was labeled with CY3 and MINPP1 was labeled with FAM. ^*^P < 0.05, ^**^P < 0.01, ^***^P < 0.001, NS: no statistics

The expression level of miRNA-30b-5p was assessed in 20 paired HBV-positive and HBV-negative HCC samples using RT-qPCR, and results showed that the expression of miRNA-30b-5p was significantly higher in HBV-positive HCC than that in HBV-negative HCC (Fig. 4a). A similar differential expression pattern of miRNA-30b-5p was observed in the HCC cell lines. In line with the MINPP1 expression (Fig. 2a), the differential expression was exhibited between the HBV-negative Huh7 cells and the HBV-positive Hep3B cells (Fig. 4b). Therefore, Huh7 and Hep3B cells were used to validate the biological function of miRNA-30b-5p. We constructed Hep3B cell lines that stably overexpress miRNA-30b-5p or were knocked down for miRNA-30b-5p (Figure S7A, B). Proliferative viability of Hep3B cells was measured by CCK-8 analysis, showing that inhibitor of miRNA-30b-5p suppressed the proliferation of Hep3B cells while miRNA-30b-5p mimics increased it (Fig. 4c). In addition, scrape motility assays revealed that inhibitor of miRNA-30b-5p suppressed the invasion ability of Hep3B cells while miRNA-30b-5p mimics promoted it (Figure S8). The oncogenic role of miRNA-30b-5p was further verified by xenograft in nude mice. It was observed that the format of tumor growth, volume, and weight from control was largely inhibited when the injected Hep3B cells were transfected with inhibitor of miRNA-30b-5p instead of miRNA-30b-5p mimics (Fig. 4d-f). HE staining and IHC analysis of PCNA in the xenograft tumor tissue demonstrated the suppression of tumor cell proliferation by inhibitor of miRNA-30b-5p (Figure S9). Moreover, FISH assay in HCC tissue samples was performed, and the result showed that unlike in HBV-negative tissue, the co-localization of miRNA-30b-5p and MINPP1 existed in HBV-positive tissue (Fig. 4g). These results confirmed that miRNA 305-5p gives a growth advantage to HBV-positive HCC cells and exerts its carcinogenic effect by inhibiting the expression of anti-oncogene MINPP1.

### miR-30b-5p/MINPP1 regulates glycolytic bypass metabolism and development of HBV-positive HCC

**Fig. 5:**
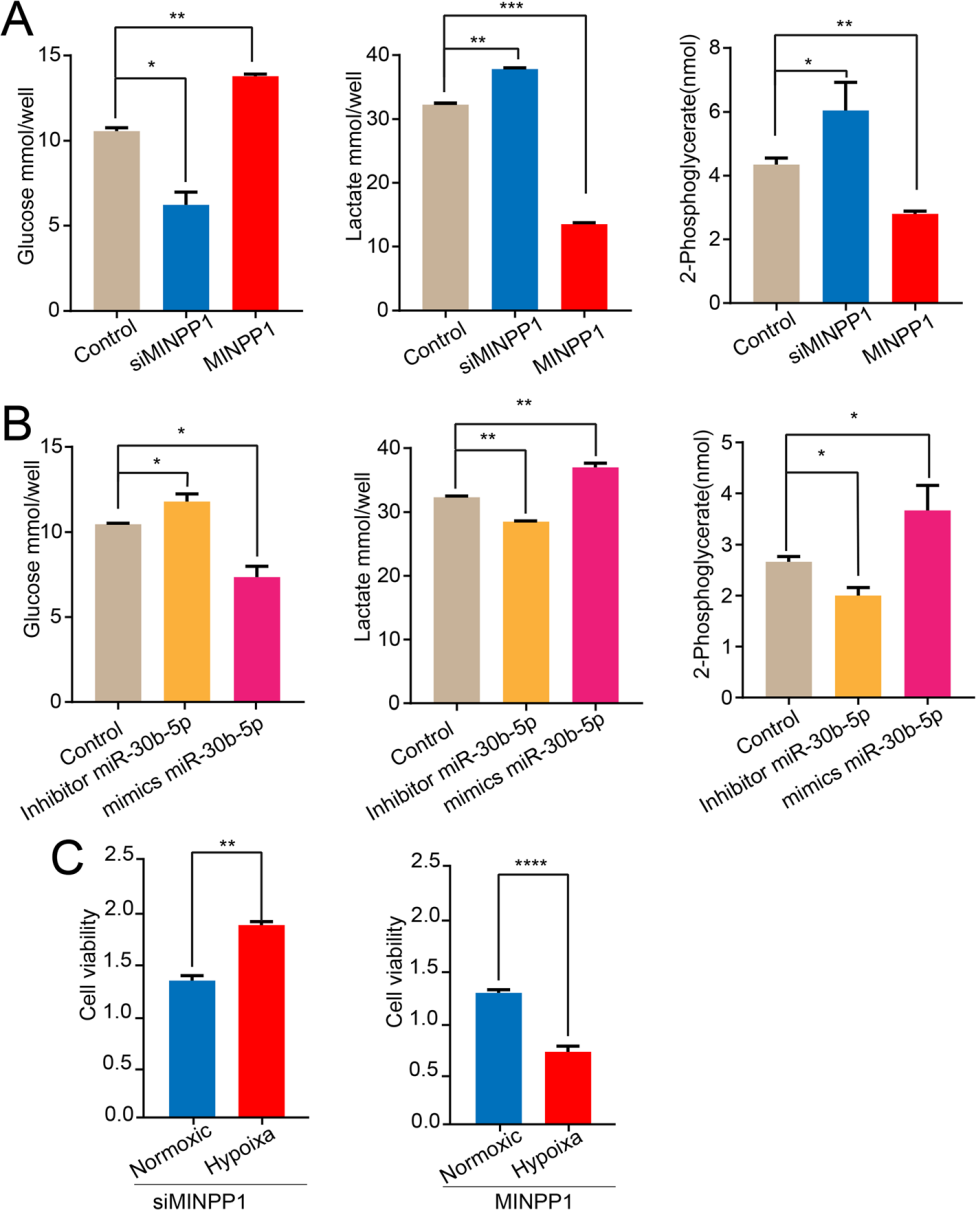
MINPP1 and miRNA-30b-5p regulate glycolytic bypass metabolism of HBV-positive HCC cells. **a** Cellular glucose (left), lactate (middle), and 2--PG (right) levels in Hep3B cell carrying MINPP1 knockdown or overexpression. **b** Cellular glucose (left), lactate (middle), and 2--PG (right) level in Hep3B cells in which miRNA-30b-5p was inhibited or overexpressed. **c** The proliferation of Hep3B cells with MINPP1 knockdown and overexpression was determined under hypoxic and normoxic conditions with CCK-8 assays. ^*^P < 0.05, ^**^P < 0.01, ^***^P < 0.001

Since MINPP1 participates in the glycolytic bypass, which is a component of the glycolysis/gluconeogenesis pathway (Figure S1), it was hypothesized that MINPP1 inhibited the development of HBV-positive HCC through a metabolic mechanism. Glycolytic bypass has two branches, the first BPGM-mediated reaction has been previously reported in numerous research (Figure S1, righ α) while the second MINPP1-dependent reaction was recently discovered (Figure S1, righ β) [13]. The glycolytic bypass is the primary negative feedback regulation of the metabolic pathway in red blood cells [16], but tumorigenesis associated with this glycolytic bypass has not been reported. For the first time, this work has preliminarily validated MINPP1 from glycolytic bypass pathway can inhibit the development of HBV-positive HCC. MINPP1 is the specific gene that regulates the catalization from 2, 3-BPG to 2-PG (Figure S1, righ β). Therefore, the relevant metabolic indicators such as glucose, lactate, and 2-PG were assessed. Overexpression of MINPP1 reduced the Hep3B cellular levels of glucose consumption, the lactate production, and the transformation from 3-PG to 2-PG (Fig. 5a). Whereas, knockdown of MINPP1 resulted in the opposite outcomes (Fig. 5a). In addition, miRNA-30b-5p mimics increased the Hep3B cellular levels of glucose consumption, the lactate production, and the transformation from 3-PG to 2-PG, while opposite results were obtained when the Hep3B cells were transfected with a inhibitor of miRNA-30b-5p (Fig. 5b). Since the glycolytic bypass is a hypoxia oxidation process, we next investigated whether the MINPP1-mediated glycolytic bypass plays an anti-oncogenetic role in hypoxic conditions. As expected, CCK-8 assays showed that knockdown of MINPP1 was more likely to promote the proliferation of Hep3B cells in hypoxia than normoxia (Fig. 5c). Whereas, overexpression of MINPP1 was more likely to inhibit the proliferation of Hep3B cells in hypoxia than normoxia (Fig. 5c). Notably, these results not only suggested a role of the glycolytic bypass as a metabolic pathway in the development of HBV-positive HCC, but also indicated that MINPP1 suppresses this tumorigenesis by inhibiting energy production via the glycolytic bypass. Meanwhile, miRNA-30b-5p, as an oncogene, is able to regulate the glycolytic bypass to produce more 2-PG for supplementing energy by modulating the expression of MINPP1.

### HBV infection as a critical factor in the development of HBV-related HCC

**Fig. 6:**
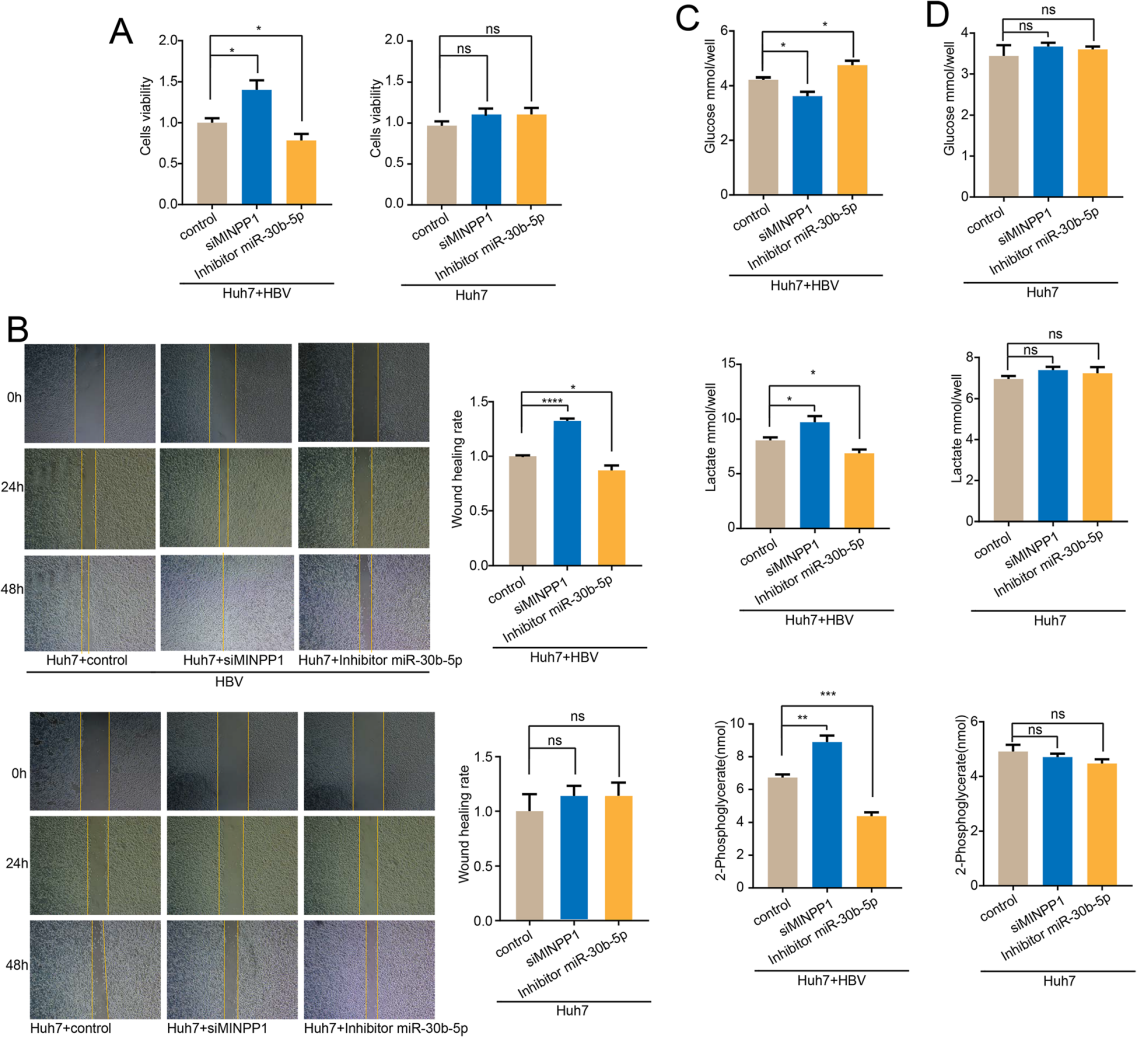
Verification of HBV infection associated with the development of HCC. **a** The proliferation of Huh7 cells transfected with pHBV1.3 (left) or not (right) with miRNA-30b-5p inhibition, MINPP1 knockdown and control was determined using the CCK-8 assay. **b** Scrape motility were measured by cell migration assays at 0, 24, and 48 h in Huh7 cells transfected with pHBV1.3 (upper) or not (down) with miRNA-30b-5p inhibition, MINPP1 knockdown and control. The wound healing rate was tested by Image J software. **c** Cellular glucose (upper), lactate (middle), and 2-PG (down) levels in Huh7 cells transfected with pHBV1.3 with miRNA-30b-5p inhibition, MINPP1 knockdown and control. **d** Cellular glucose (upper), lactate (middle), and 2--PG (down) levels were measured in the Huh7 cells transfected with inhibitor of miRNA-30b-5p, MINPP1 knockdown, and control construct. ^*^P < 0.05, ^**^P < 0.01, ^***^P < 0.001, ^****^P < 0.0001, NS: no statistics

Since HBV infection is a process of interplay with HBV-DNA, host genome, and nocoding RNA modulation to promote the development of HCC [2]. We speculated that HBV infection may play a critical role in regulating MINPP1 and miRNA-30b-5p to either inhibit or promote the HBV-positive HCC tumorigenesis. Thus, the HBV-negative Huh7 cells were transfected with pHBV1.3 conatining complete HBV genome and control empty vector (EV) (Figure S10) to perform the following experiments. The expression of MINPP1 and miRNA-30b-5p was significantly down-regulated and upregulated, respectively, in Huh7 cells transfected with pHBV1.3 respectively compared to control EV (Figure S11). CCK-8 assays showed that, in contrast to the control, knockdown of MINPP1 promoted the proliferative viability of Huh7 cells transfected with pHBV1.3 (Fig. 6a, left). We could also observed that inhibitor of miRNA-30b-5p suppressed the proliferative viability of Huh7 cells transfected with pHBV1.3 (Fig. 6a, left). Nonetheless, this difference was not observed in Huh7 cells without pHBV1.3 transfection (Fig. 6a, right). In addition, similar results were obtained in scrape motility assays of the Huh7 cells transfected with pHBV1.3 but not the untransfected Huh7 cells (Fig. 6b). We next investigated whether the involvement of HBV infection in the glycolytic bypass contributes to the development of HBV-positive HCC. The Huh7 cells transfected with pHBV1.3 displayed an increment of glucose consumption as well as a raised production of lactate and 2-PG (Figure S12). It was also observed that either knockdown of MINPP1 or inhibitor of miRNA-30b-5pinfluenced the metabolic indicators such as glucose consumption and the production of lactate and 2-PG in Huh7 cells transfected with pHBV1.3 (Fig. 6c), while this influence was not displayed in the untransfected Huh7 cells (Fig. 6d). All the above results suggested that HBV infection regulates the development of HBV-positive HCC, and that the miRNA-30b-5p/MINPP1 contribution to HCC through the glycolytic bypass was limited to HBV-related HCC.

### HBV promotes miRNA-30b-5p expression through HBp interaction with FOXO3

**Fig. 7:**
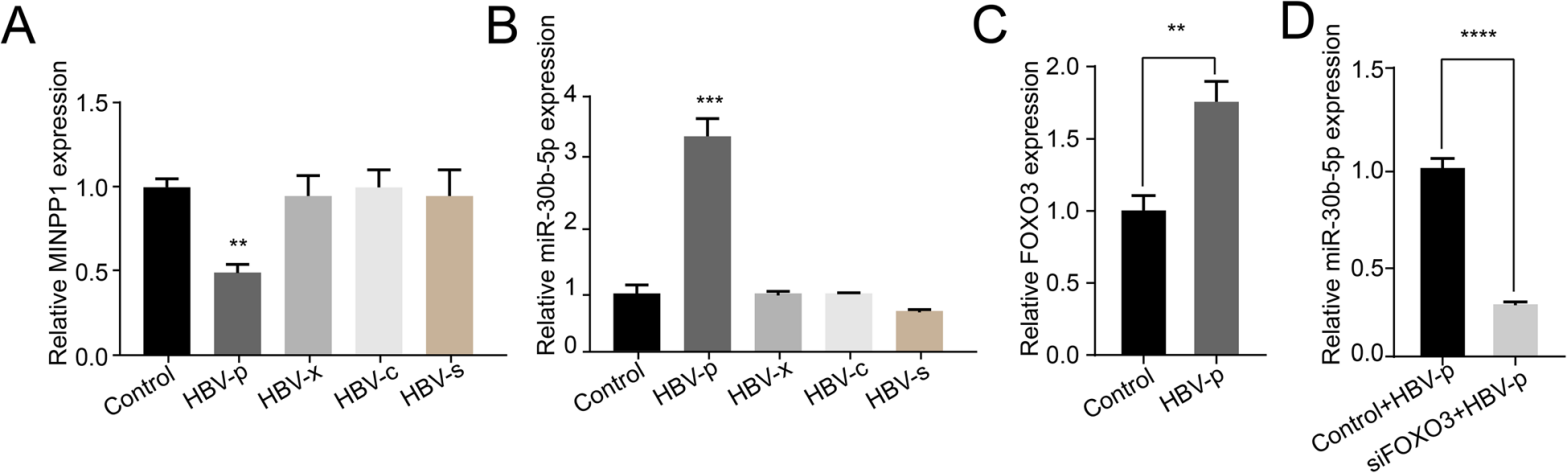
Identification of HBp as a key factor that activates miRNA-30b-5p/MINPP1/glycolytic bypass axis in HBV-positive HCC. The expression level of (**a**) MINPP1 and (**b**) miRNA-30b-5p was measured by RT-qPCR after plasmids expressing viral proteins HBx, HBs, HBp, and HBc were transfected in Huh7 cells. **c** FOXO3 expression in Huh7 cells co-transfected with HBp was measured with RT-qPCR assay. **d** RT-qPCR assay was used to measure miRNA-30b-5p expression in Huh7 cells co-transfected with HBp and FOXO3 siRNAs. ^**^P < 0.01, ^***^P < 0.001, ^****^P < 0.0001

It was reported that the expression of miRNA could be regulated by a HBV protein [8]. Therefore, the up-regulated miRNA-30b-5p and down-regulated MINPP1 were validated by introducing the plasmid-expressing HBV proteins HBx, HBs, HBp, and HBc into the HBV-negative Huh7 cells. The results showed that only HBp significantly down-regulated the expression of MINPP1 and up-regulated the expression of miRNA-30b-5p (Fig. 7a, b). Previously, studies reported that HBV protein HBx indirectly regulates the expression of miRNA through its interactional transcriptional factors (TF) [22]. For that reason, we hypothesized that HBp promoted the expression of miRNA-30b-5p by interacting with a TF. Previous studies proved that the TF of FOXO3 was a candidate target for miR-30b and there was direct interaction between FOXO3 and miR-30b [23]. And accumulating evidence has shown that the role of Forkhead box O (FOXO) family of TF (including FOXO3) in oncogenesis [24]. Besides, the post-transcriptional regulation of FOXO3a activity was modulated by miRNA [25]. Thus, we tested whether HBp influenced the expression of miRNA-30b-5p through FOXO3. The Huh7 cells transfected with plasmid expressing HBp (Figure S13) displayed the significantly elevated expression of FOXO3 compared to the cells without HBp (Fig. 7c). However, the up-regulated miRNA-30b-5p expression through introducing HBp into Huh7 cells could be significantly blocked by knockdown of FOXO3 (Fig. 7d). The above results indicated that HBp is a key molecule in initiating the miRNA-30b-5p/MINPP1 axis in HBV-related HCC pathogenesis, and that it can promote the expression of miRNA-30b-5p through its interaction with FOXO3.

### The clinical value of MINPP1 in HBV-positive HCC

**Fig. 8:**
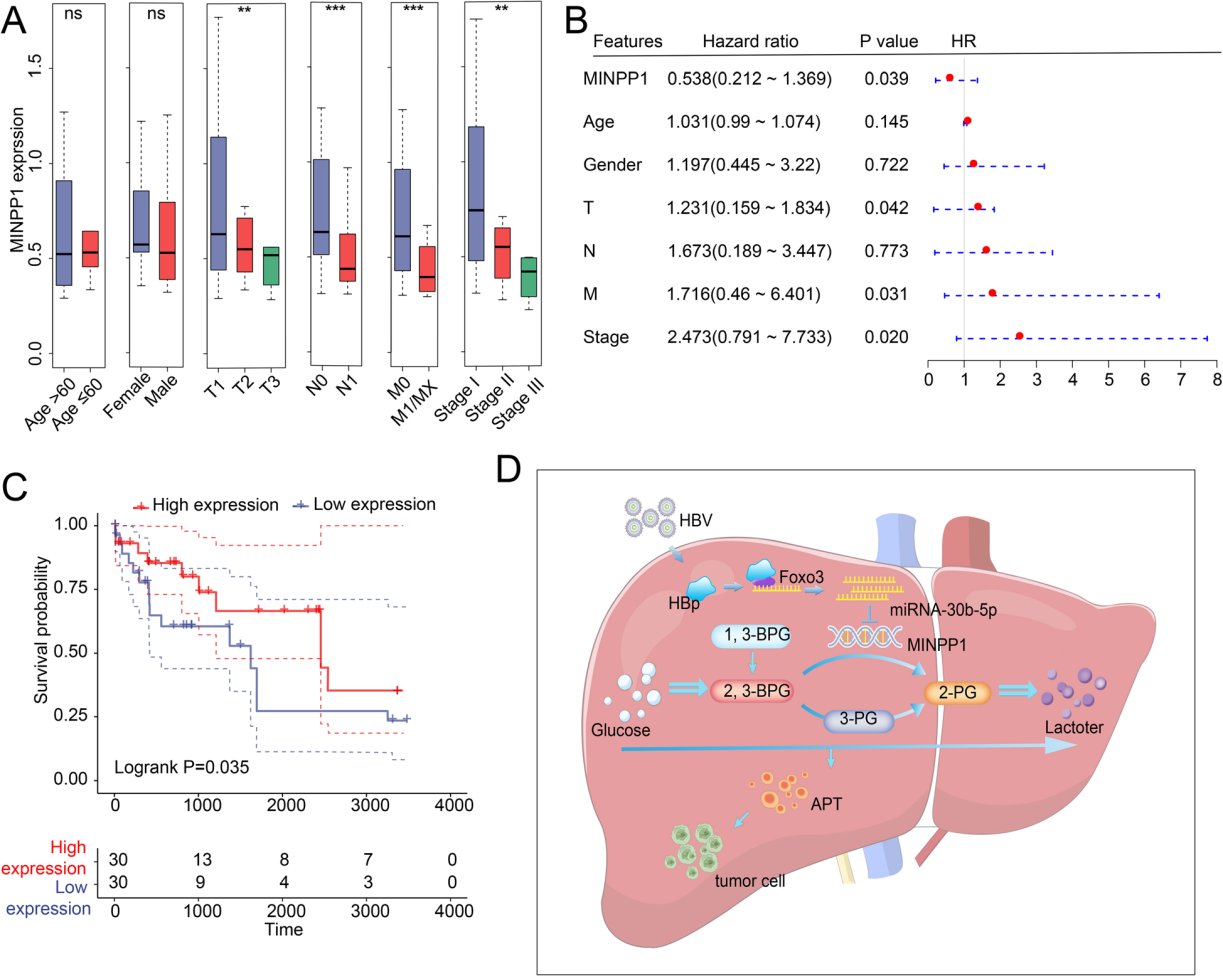
The clinical value of MINPP1 was tested by bioinformatic analysis. **a** The association of MINPP1 with HBV-positive HCC clinical and pathological stages was verified in a HCC cohort from the TCGA database. **b** A Forest plot was plotted to present the results of Cox regression analysis conducted to show the clinical information and MINPP1 for survival in the TCGA database. **c** KM analysis of the survival of high and low MINPP1expression groups. **d** Schematic illustration of the HBp/FOXO3/miRNA-30b-5p/MINPP1 axis that promotes HBV-positive HCC development through the glycolytic bypass. ^**^P < 0.01, ^***^P < 0.001, ^****^P < 0.0001, NS: No statistics

This study first revealed that MINPP1 is involved in the pathogenesis of HBV-positive HCC. Using bioinformatics analysis, the clinical value of MINPP1 was validated in a HCC cohort from the TCGA database. The expression level of MINPP1 was negatively associated with the clinicopathological stages, while having no relation with age and gender (Fig. 8a). When the MINPP1 level was treated as a continuous variable in the Cox regression model, the forest plot showed that it remained to be an independent factor (*P* = 0.039) (Fig. 8b). Hazard Ratio (HR) < 1 indicated that MINPP1 was a favorable factor for HBV-positive HCC (Fig. 8b). KM analysis showed that high expression of MINPP1 displayed longer survival time than low expression of MINPP1 (Fig. 8c). These findings suggested that MINPP1 is a potentially clinical biomarker for HBV-positive HCC development. Taken together, this study suggested a HBV-related HCC mechanism involving the HBp/FOXO3/miRNA-30b-5p/MINPP1 axis via the glycolytic bypass. In this proposed model, HBV infection promotes the expression of miRNA-30b-5p through HBp interaction with TF FOXO3. The induced miRNA-30b-5p then suppresses the expression of MINPP1 which is involved in the glycolytic bypass. The inhibited MINPP1-dependent glycotic bypass facilitates the transformation from 3-PG to 2-PG geared towards providing more energy for the development of HBV-positive HCC (Fig. 8d).

## Discussion

HBV is the most common causative factor for HCC. HBV infections can lead to chronic hepatitis and cirrhosis [26]. Therefore, understanding of the molecular mechanisms through which HBV triggers HCC is crucial for effective HCC diagnosis and management. In this study, microarray, sequencing, cell biology experiment, and multi-dimensional bioinformatics methods were employed to analyze interactions among genes, miRNA, signaling pathways that regulate the progression of HBV-related HCC. Unlike other studies that compared different molecules between tumor cells and normal cells, we compared HBV-positive and HBV-negative HCC tissue or cells. In this way, we precisely reveal the molecular mechanisms through which HBV infection causes HCC.

In this study, 110 genes were found to be significantly differentially expressed between HBV-positive and HBV-negative HCC liver tissues, which was far less than the number often reporeted between HCC and normal tissues [27–29]. Moreover, 6 biological pathways identified to be associated with these differentially expressed genes. Therefore, our results reveal shared and uniques pathways between HBV-positive and -negative HCC allowed to focus shared pathogenesis pathways and evidenced those involved uniquely in the HBV pro-cancer activity. Among the 6 biological pathways, glycolysis/gluconeogenesis was found to be closely associated with metabolism that drives tumorigenesis [30]. In addition, we identify the MINPP1 gene which participate in the glycolytic bypass, a component of anaerobic glycolysis. These findings are in agreeement with previous studies, which reported that tumor metabolic reprogramming mechanisms provide energy for its own growth through glycolysis with relatively low oxidative productivity [10]. Several genes involved in metabolism, such as *TUG1* [31], *HIF* [32], and *PKM2* [33] regulate the development of HCC. However, there is no evidence that metabolic pathways involved in tumorigenesis contribute to dysregulation of MINPP1. Thus, exploratory experiments were performed to determine the function of MINPP1 in HCC. Results showed that MINPP1 inhibits HCC development, but this was limited to HBV-positive HCC and not other liver cancers. These findings reveal that MINPP1 expression is decreased in HBV-positive HCC, suggesting MINPP1 may be a specific biomarker of HBV-positive HCC. MINPP1 enzyme hydrolzyes inositol pentakisphosphate (IP5) and inositol hexakisphosphate (IP6) [13]. Previous studies found that IP6 is a co-factor required for efficient production of infectious human immunodeficiency virus (HIV) particles [34]. Clifton et al. found that MINPP1 can ablate IP6, therefore decrease the HIV infectious particles, whereas a decrease in MINPP1 expression would increase IP6 expression favoring the formation of HIV particles [35]. We hypothsized that MINPP1 may suppressthe development of HBV-positive HCC through other metabolic pathways, such as ablation of IP6 which inhibits HBV formation and replication. This may be a novel hypothesis to the mechanism of this reseach, which worthy of further research.

This study demonstrates a novel pathomechanism of HCC tumorigenesis involving the glycolytic bypass (Figure S1, right α), which is a separate dephosphorylation of 2,3-BPG to 2-PG, a process catalyzed by MINPP1 [13]. Most of the molecules studied in glycolytic bypass regulate systemic oxygen homeostasis [36]. In most mammals, 2,3-BPG of the glycolytic bypass is the major allosteric effector which facilitate the release of oxygen from hemoglobin in red blood cells to the surrounding tissue [37]. Therefore, the glycolytic bypass facilitates the supply of oxygen is thus a fundamental physiological negative feedback regulation for hypoxia [14]. This study suggests for the first time that tumor cells may mimic the glucose metabolism of red blood cells through the glycolytic bypass. This is a novel approach for studying the metabolism of HBV-positive HCC. It is also important to investigate whether the glycolytic bypass participates in other types of cancer.

Studies have reported that gene involved in diseases interact with other molecules or are regulated by upstream molecules or modulated by epigenetic modification [38, 39]. We therefore aimed to identify molecules that regulate the expression of MINPP1. Research on functions of non-coding RNAs has achieved significant results in recent years, especially the regulatory mechanisms of miRNA [40]. In addition, several studies have revealed that miRNA participates in tumorigenesis, including HCC development [41]. Here, we found thatmiRNA-30b-5p was up-regulated in HBV-positive HCC and inhibited the translation of MINPP1 by binding to its base binding site. Further analysis revealed that miRNA-30b-5p could be a potential therapeutic target in HBV-positive HCC.

We combined experimental and bioinformatics analysis methods in this research. Multi-dimensional bioinformatic analysis was conducted to identify potential biomarkers of gene and miRNA, and their association in HBV-positive HCC. Importantly, through bioinformatic analysis, we analyzed the expression level of MINPP1 and its clinical significance in a big database of HCC samples. Bioinformatic analysis based on big data transcriptome conquers the shortcomings of small sample size and low homogeneity of tissue, and confirms the plausibility of accurately identifying the pathogenesis and related biomarker of the disease [42]. By combining bioinformatics and experimental analyses, we obtained more accurate results.

The miRNA-30b-5p/MINPP1 axis contributes to HCC development through the glycolytic bypass, and this is limited to HBV-positive HCC. The present study shows that HBV infection activates the miRNA-30b-5p/MINPP1/glycolytic bypass axis in HBV-positive HCC. Furthermore, we show that HBp promotes the expression of miRNA-30b-5p. Emerging evidence reveals that miRNAs are involved in HBV replication [43]. Such studies have also revealed the mechanism through which HBV modulates expression of cellular miRNAs, such as intercation between transcriptional factors and promoters [44] and epigenetic modifications of promoters by HBV proteins [45]. In this study, we found that HBp promotes the expression of miRNA-30b-5p by interacting with FOXO3. HBp is a broad-range transactivator that stimulates the transcription of its own HBV genes as well as other host genes, including proto-oncogenes related to HCC. Several studies have investigated how HBx regulates miRNAs, but few have explored how HBp modulates miRNAs. Therefore, further investigations are essential to accurately reveal how HBp regulate miRNA-30b-5p.

## Conclusion

In this study, we uncovered a novel tumorigenesis axis, namely HBp/FOXO3/miRNA-30b-5p/MINPP1 which promotes the progression of HBV-positive HCC through glycolytic bypass and is limited to HBV-related HCC. Moreover, we highlighted the MINPP1 gene as a robust biomarker of HBV-positive HCC. Our study also provided novel insights to advance the knowledge on the pathogenesis of HBV-positive HCC from the perspective of the metabolic mechanisms and potential biomarkers for clinical application in HCC diagnosis and treatment.

## Supplementary Information

**Additional file 1: Figure S1.** Left: the original graph of glycolysis/Gluconeogenesis pathway obtained from the KEGG website (https://www.kegg.jp/). We were granted publication permission from Kanehisa Laboratories, Japan. Right: The glycolytic bypass is a component of the glycolysis/gluconeogenesis pathway, which contains two branches, (α) one is well recognized, and (β) the other has recently been discovered.

**Additional file 2: Figure S2.** Measure on transfection efficiency of PGLV3/H1/GFP lentiviral and siRNA of MINPP1. Relative MINPP1 expression levels after transfection with (A) PGLV3/H1/GFP lentiviral and (B) siRNA in Hep3B cells. (C) The image of cell before transfected with PGLV3/H1/GFP lentiviral of MINPP1. (D) The transfection efficiency of PGLV3/H1/GFP lentiviral of MINPP1 was measured by green fluorescence intensity from GFP. ^**^P < 0.01, ^****^P < 0.0001.

**Additional file 3: Figure S3.** Results of HE staining and IHC analysis for PCNA in xenograft tumor tissues obtained from nude mice treated with Hep3B cells overexpressing MINPP1 and control.

**Additional file 4: Figure S4.** Validation of biological function of MINPP1 in the database. WGCNA algorithm was used to analyze the gene expression profiles related to MINPP1 in (A) GSE55092 and (B) TCGA cohorts. (C) GO analysis of the association between genes and biological processes. (D) KEGG analysis of genes involved in pathways.

**Additional file 5: Figure S5.** miRNAs differentially expressed between HBV-positive and HBV-negative HCC tissues. (A) Volcano Plot showing the differentially expressed miRNAs between HBV-positive and HBV-negative HCC tissues. (B) heatmap clustering of the up-regulated miRNAs in HBV-positive and HBV-negative tissues.

**Additional file 6: Figure S6.** The association between MINPP1 and miRNA-30b-5p was validated in the database. (A) Correlation between MINPP1 and predicted miRNAs was analyzed in the TCGA cohort. (B) The interaction network of MINPP1 and the predicted miRNA.

**Additional file 7: Figure S7.** Measure on transfection efficiency of inhibitors and mimics of miRNA-30b-5p. The transfection efficiency of miRNA-30b-5p in Hep3B cells. The relative expression levels of miRNA-30b-5p after transfection with (A) inhibitors and (B) mimics in Hep3B cells. (C) The image of cell before transfected with inhibitors of miRNA-30b-5p. (D) The transfection efficiency of inhibitors of miRNA-30b-5p was measured by green fluorescence intensity from GFP. ^***^P < 0.001.

**Additional file 8: Figure S8.** Scrape motility were measured by cell migration assays at 0, 24, and 48 h in the Hep3B cells with miRNA-30b-5p inhibition and overexpression. The wound healing rate was tested by Image J software.

**Additional file 9: Figure S9.** Results of HE staining and IHC analysis of PCNA in xenograft tumors from nude mice injected with Hep3B cells treated with the inhibitor of miRNA-30b-5p and control.

**Additional file 10: Figure S10.** Relative expression levels of HBV after transfection with HBV DNA (pHBV1.3) into Huh7 cells. ^****^P < 0.0001.

**Additional file 11: Figure S11.** The expression level of MINPP1 (left) and miRNA-30b-5p (right) was examined by RT-qPCR after the Huh7 cells were transfected with pHBV1.3 or control. ^*^P < 0.05, ^****^P < 0.0001.

**Additional file 12: Figure S12.** Cellular glucose (left), lactate (middle), and 2-PG (right) levels were measured in the Huh7 cells transfected with pHBV1.3 and control empty vector (EV). ^**^P < 0.01, ^***^P < 0.001.

**Additional file 13: Figure S13.** Measure on transfection efficiency of viral proteins HBp plasmids. (A) Relative expression levels of HBp after transfection with viral proteins HBp plasmids into Huh7 cells. (B) The image of cell before transfected with viral proteins HBp plasmids. (C) The transfection efficiency of viral proteins HBp plasmids was measured by green fluorescence intensity from GFP. ^****^P < 0.0001.

**Additional file 14: Table S1.** The clinical characteristic of HBV-positive and HBV-negative HCC patient using in the microarray analysis. **Table S2.** The primers for RT-qPCR and sequences using in this study. **Table S3.** Different expression mRNAs between HBV-positive and HBV-negative HCC samples. **Table S4.** Different expression miRNAs between HBV-positive and HBV-negative HCC samples.

## Data Availability

The datasets used in this study were uploaded to GEO database.

## Abbreviations

HBV: Hepatitis B virus
HCC: Hepatocellular carcinoma
2,3-BPG: 2,3-bisphosphoglycerate
HBp: HBV protein P
HBs: HBV protein S
HBc: HBV protein C
HBx: HBV protein X
miRNAs: microRNAs
mRNA: messenger RNA
1,3-BPG: 1,3-bisphosphoglycerate
3-PG: 3-phosphoglycerate
2-PG: 2-phosphoglycerate
ARDS: acute respiratory distress syndrome
BPGM: 2,3-PG synthase/2-phosphatase
MINPP1: Multiple inositol-polyphosphate phosphatase 1
FOXO3: Forkhead Box O3
RNA-Seq: RNA sequencing
GEO: Gene Expression Omnibus database
WGCNA: Weighted Correlation Network Analysis
KEGG: Kyoto Encyclopedia of Genes and Genomes
KM: Kaplan–Meier
GO: Gene ontology
WT: Wild type
MUT: Mutation of the binding site

## References

[1] Wong MCS, Huang JLW, George J, Huang J, Leung C, Eslam M (2019). The changing epidemiology of liver diseases in the Asia-Pacific region. Nat Rev Gastroenterol Hepatol.

[2] Levrero M, Zucman-Rossi J (2016). Mechanisms of HBV-induced hepatocellular carcinoma. J Hepatol.

[3] Tong S, Revill P. Overview of hepatitis B viral replication and genetic variability. J Hepatol. 2016;64(1 Suppl):S4–s16. 10.1016/j.jhep.2016.01.027 PMC4834849 27084035

[4] Hu J, Protzer U, Siddiqui A. Revisiting Hepatitis B Virus: Challenges of Curative Therapies. J Virol. 2019;93(20):e01032–19. 10.1128/JVI.01032-19 PMC6798116 31375584

[5] Lucifora J, Arzberger S, Durantel D, Belloni L, Strubin M, Levrero M (2011). Hepatitis B virus X protein is essential to initiate and maintain virus replication after infection. J Hepatol.

[6] Oropeza CE, Tarnow G, Sridhar A, Taha TY, Shalaby RE, McLachlan A (2020). The regulation of HBV transcription and replication. Adv Exp Med Biol.

[7] Zhang ZH, Wu CC, Chen XW, Li X, Li J, Lu MJ (2016). Genetic variation of hepatitis B virus and its significance for pathogenesis. World J Gastroenterol.

[8] Sartorius K, Makarova J, Sartorius B, An P, Winkler C, Chuturgoon A, et al. The Regulatory Role of MicroRNA in Hepatitis-B Virus-Associated Hepatocellular Carcinoma (HBV-HCC) Pathogenesis. Cells. 2019;8(12):1504. 10.3390/cells8121504 PMC6953055 31771261

[9] Potter M, Newport E, Morten KJ (2016). The Warburg effect: 80 years on. Biochem Soc Trans.

[10] Lu J (2019). The Warburg metabolism fuels tumor metastasis. Cancer Metastasis Rev.

[11] Shankaraiah RC, Veronese A, Sabbioni S, Negrini M (2018). Non-coding RNAs in the reprogramming of glucose metabolism in cancer. Cancer Lett.

[12] Subramaniam S, Jeet V, Clements JA, Gunter JH, Batra J (2019). Emergence of MicroRNAs as key players in Cancer cell metabolism. Clin Chem.

[13] Cho J, King JS, Qian X, Harwood AJ, Shears SB (2008). Dephosphorylation of 2,3-bisphosphoglycerate by MIPP expands the regulatory capacity of the Rapoport-Luebering glycolytic shunt. Proc Natl Acad Sci U S A.

[14] Kauffman KJ, Pajerowski JD, Jamshidi N, Palsson BO, Edwards JS (2002). Description and analysis of metabolic connectivity and dynamics in the human red blood cell. Biophys J.

[15] Rapoport I, Berger H, Elsner R, Rapoport S (1977). PH-dependent changes of 2,3-bisphosphoglycerate in human red cells during transitional and steady states in vitro. Eur J Biochem.

[16] van Wijk R, van Solinge WW (2005). The energy-less red blood cell is lost: erythrocyte enzyme abnormalities of glycolysis. Blood..

[17] Kanehisa M, Furumichi M, Tanabe M, Sato Y, Morishima K (2017). KEGG: new perspectives on genomes, pathways, diseases and drugs. Nucleic Acids Res.

[18] Rosvall M, Bergstrom CT (2010). Mapping change in large networks. PLoS One.

[19] Chen R, Zhu S, Fan XG, Wang H, Lotze MT, Zeh HJ (2018). High mobility group protein B1 controls liver cancer initiation through yes-associated protein -dependent aerobic glycolysis. Hepatology (Baltimore, Md).

[20] Guo W, Qiu Z, Wang Z, Wang Q, Tan N, Chen T (2015). MiR-199a-5p is negatively associated with malignancies and regulates glycolysis and lactate production by targeting hexokinase 2 in liver cancer. Hepatology (Baltimore, Md).

[21] Fabian MR, Sonenberg N, Filipowicz W (2010). Regulation of mRNA translation and stability by microRNAs. Annu Rev Biochem.

[22] Xu X, Fan Z, Kang L, Han J, Jiang C, Zheng X (2013). Hepatitis B virus X protein represses miRNA-148a to enhance tumorigenesis. J Clin Invest.

[23] Duan ZQ, Li Y, Li L (2017). Experimental evidences for miR-30b as a negative regulator of FOXO3 upregulated by kynurenine. Immunol Res.

[24] Deng Y, Wang F, Hughes T, Yu J (2018). FOXOs in cancer immunity: Knowns and unknowns. Semin Cancer Biol.

[25] Liu Y, Ao X, Ding W, Ponnusamy M, Wu W, Hao X (2018). Critical role of FOXO3a in carcinogenesis. Mol Cancer.

[26] Mysore KR, Leung DH (2018). Hepatitis B and C. Clin Liver Dis.

[27] Xu XR, Huang J, Xu ZG, Qian BZ, Zhu ZD, Yan Q (2001). Insight into hepatocellular carcinogenesis at transcriptome level by comparing gene expression profiles of hepatocellular carcinoma with those of corresponding noncancerous liver. Proc Natl Acad Sci U S A.

[28] Okamoto M, Utsunomiya T, Wakiyama S, Hashimoto M, Fukuzawa K, Ezaki T (2006). Specific gene-expression profiles of noncancerous liver tissue predict the risk for multicentric occurrence of hepatocellular carcinoma in hepatitis C virus-positive patients. Ann Surg Oncol.

[29] Okabe H, Satoh S, Kato T, Kitahara O, Yanagawa R, Yamaoka Y (2001). Genome-wide analysis of gene expression in human hepatocellular carcinomas using cDNA microarray: identification of genes involved in viral carcinogenesis and tumor progression. Cancer Res.

[30] DeBerardinis RJ, Chandel NS (2016). Fundamentals of cancer metabolism. Sci Adv.

[31] Lin YH, Wu MH, Huang YH, Yeh CT, Cheng ML, Chi HC (2018). Taurine up-regulated gene 1 functions as a master regulator to coordinate glycolysis and metastasis in hepatocellular carcinoma. Hepatology (Baltimore, Md).

[32] Zhang X, Li Y, Ma Y, Yang L, Wang T, Meng X (2018). Yes-associated protein (YAP) binds to HIF-1α and sustains HIF-1α protein stability to promote hepatocellular carcinoma cell glycolysis under hypoxic stress. J Exp Clin Cancer Res.

[33] Xu Q, Tu J, Dou C, Zhang J, Yang L, Liu X (2017). HSP90 promotes cell glycolysis, proliferation and inhibits apoptosis by regulating PKM2 abundance via Thr-328 phosphorylation in hepatocellular carcinoma. Mol Cancer.

[34] Dick RA, Mallery DL, Vogt VM, James LC. IP6 Regulation of HIV Capsid Assembly, Stability, and Uncoating. Viruses. 2018;10(11):640. 10.3390/v10110640 PMC6267275 30445742

[35] Ricana CL, Lyddon TD, Dick RA, Johnson MC (2020). Primate lentiviruses require inositol hexakisphosphate (IP6) or inositol pentakisphosphate (IP5) for the production of viral particles. PLoS Pathog.

[36] Yoshida T, Prudent M, D'Alessandro A (2019). Red blood cell storage lesion: causes and potential clinical consequences. Blood Transfus.

[37] Benesch R, Benesch RE, Yu CI (1968). Reciprocal binding of oxygen and diphosphoglycerate by human hemoglobin. Proc Natl Acad Sci U S A.

[38] Corella D, Ordovas JM (2017). Basic concepts in molecular biology related to genetics and epigenetics. Rev Esp Cardiol (Engl Ed).

[39] Rao CV, Asch AS, Yamada HY (2017). Frequently mutated genes/pathways and genomic instability as prevention targets in liver cancer. Carcinogenesis..

[40] Pritchard CC, Cheng HH, Tewari M (2012). MicroRNA profiling: approaches and considerations. Nat Rev Genet.

[41] Di Leva G, Garofalo M, Croce CM (2014). MicroRNAs in cancer. Annu Rev Pathol.

[42] Gligorijević V, Malod-Dognin N, Pržulj N (2016). Integrative methods for analyzing big data in precision medicine. Proteomics..

[43] Fan HX, Tang H (2014). Complex interactions between microRNAs and hepatitis B/C viruses. World J Gastroenterol.

[44] Wang J, Chen J, Liu Y, Zeng X, Wei M, Wu S (2019). Hepatitis B Virus Induces Autophagy to Promote its Replication by the Axis of miR-192-3p-XIAP Through NF kappa B Signaling. Hepatology (Baltimore, Md).

[45] Xie KL, Zhang YG, Liu J, Zeng Y, Wu H (2014). MicroRNAs associated with HBV infection and HBV-related HCC. Theranostics..

