# Supplementary figures and images for: Hepatitis B virus P protein initiates glycolytic bypass in HBV-related hepatocellular carcinoma via a FOXO3/miRNA-30b-5p/MINPP1 axis
Source: J Exp Clin Cancer Res. 2021 Jan 4;40:1. doi: 10.1186/s13046-020-01803-8 (PMC7779247; doi:10.1186/s13046-020-01803-8)

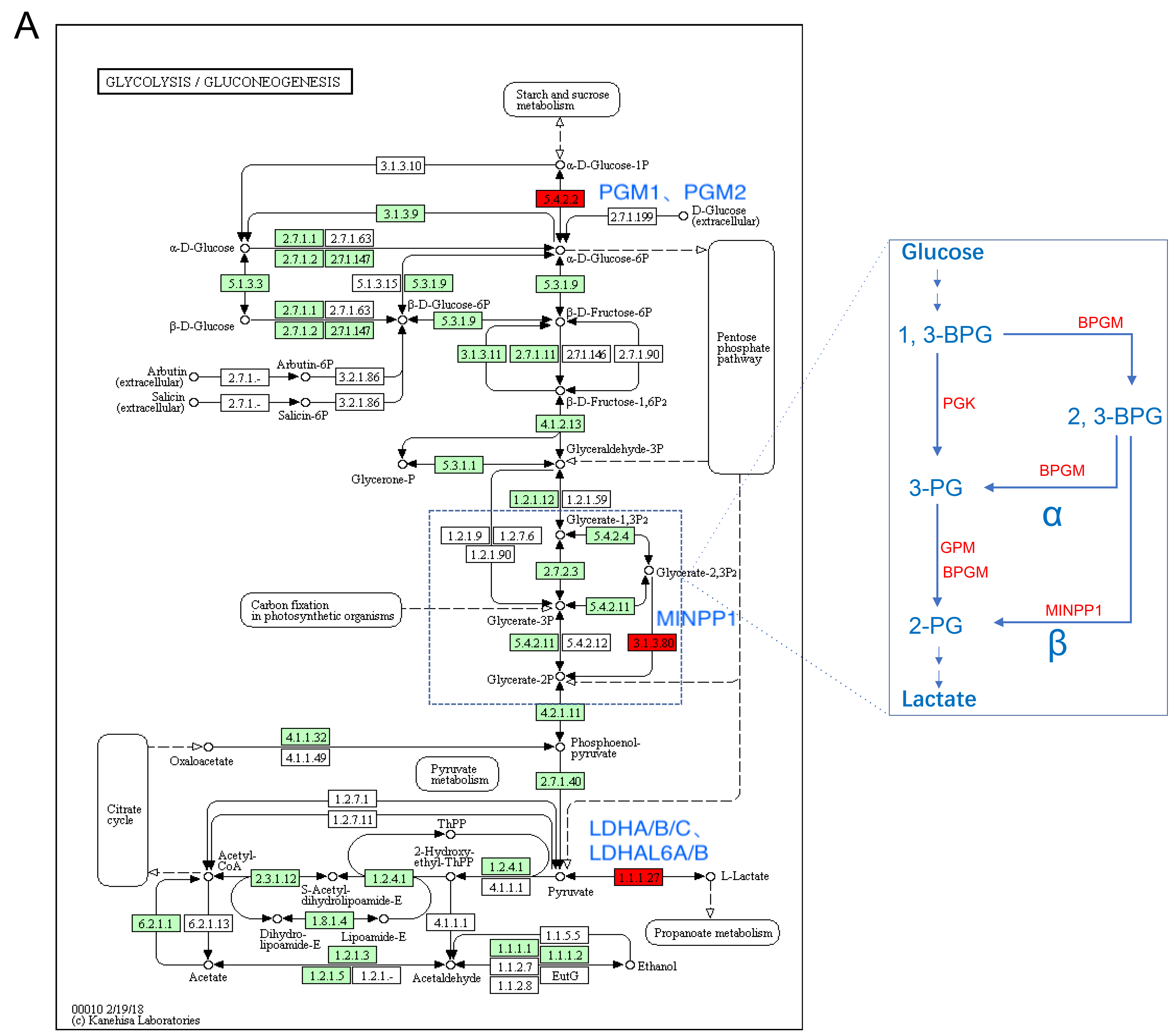

Supplement: Supplementary file 1 — Additional file 1: Figure S1. Left: the original graph of glycolysis/Gluconeogenesis pathway obtained from the KEGG website (https://www.kegg.jp/). We were granted publication permission from Kanehisa Laboratories, Japan. Right: The glycolytic bypass is a component of the glycolysis/gluconeogenesis pathway, which contains two branches, (α) one is well recognized, and (β) the other has recently been discovered. [file 13046_2020_1803_MOESM1_ESM.tif]

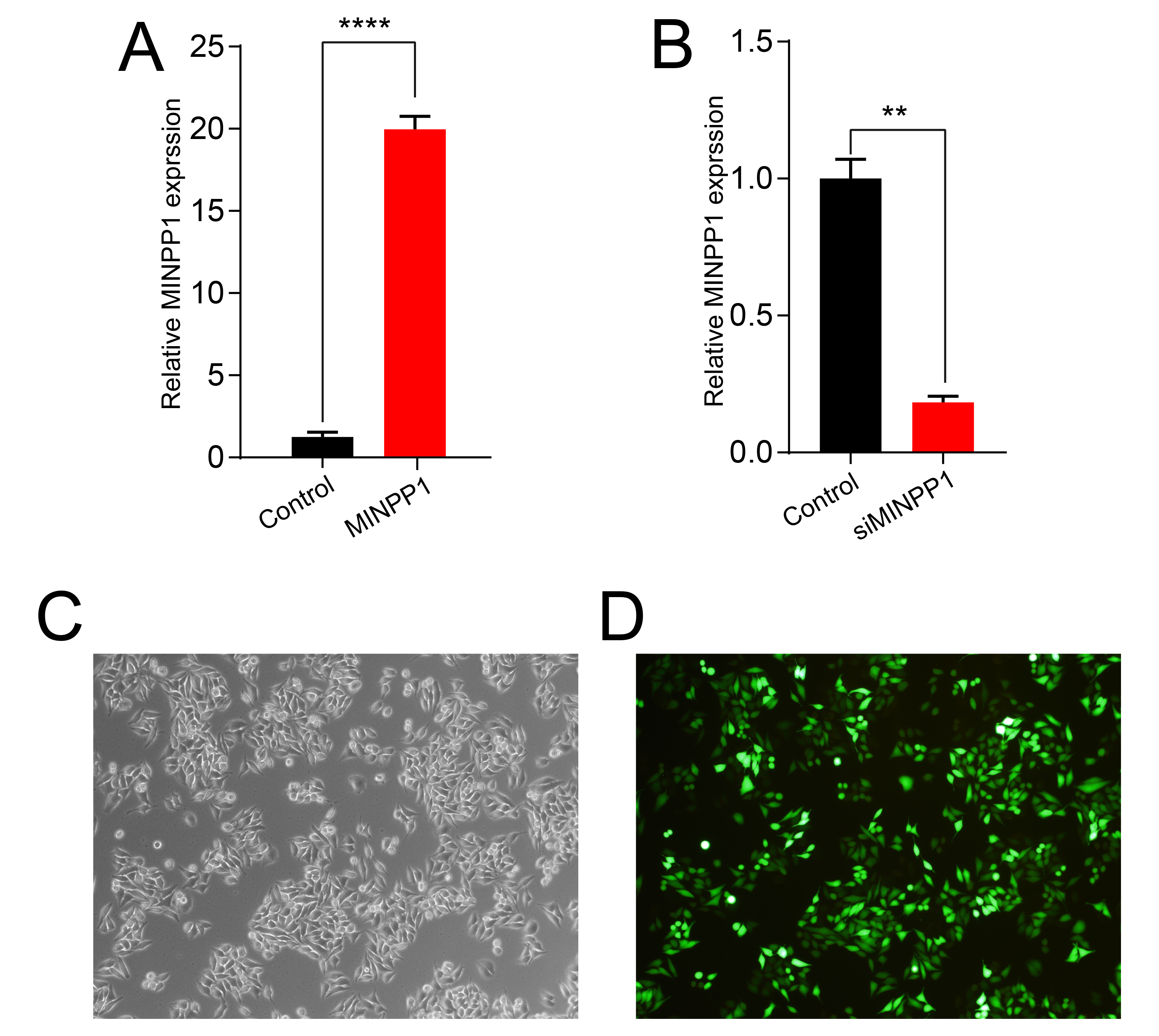

Supplement: Supplementary file 2 — Additional file 2: Figure S2. Measure on transfection efficiency of PGLV3/H1/GFP lentiviral and siRNA of MINPP1. Relative MINPP1 expression levels after transfection with (A) PGLV3/H1/GFP lentiviral and (B) siRNA in Hep3B cells. (C) The image of cell before transfected with PGLV3/H1/GFP lentiviral of MINPP1. (D) The transfection efficiency of PGLV3/H1/GFP lentiviral of MINPP1 was measured by green fluorescence intensity from GFP. **P < 0.01, ****P < 0.0001. [file 13046_2020_1803_MOESM2_ESM.tif]

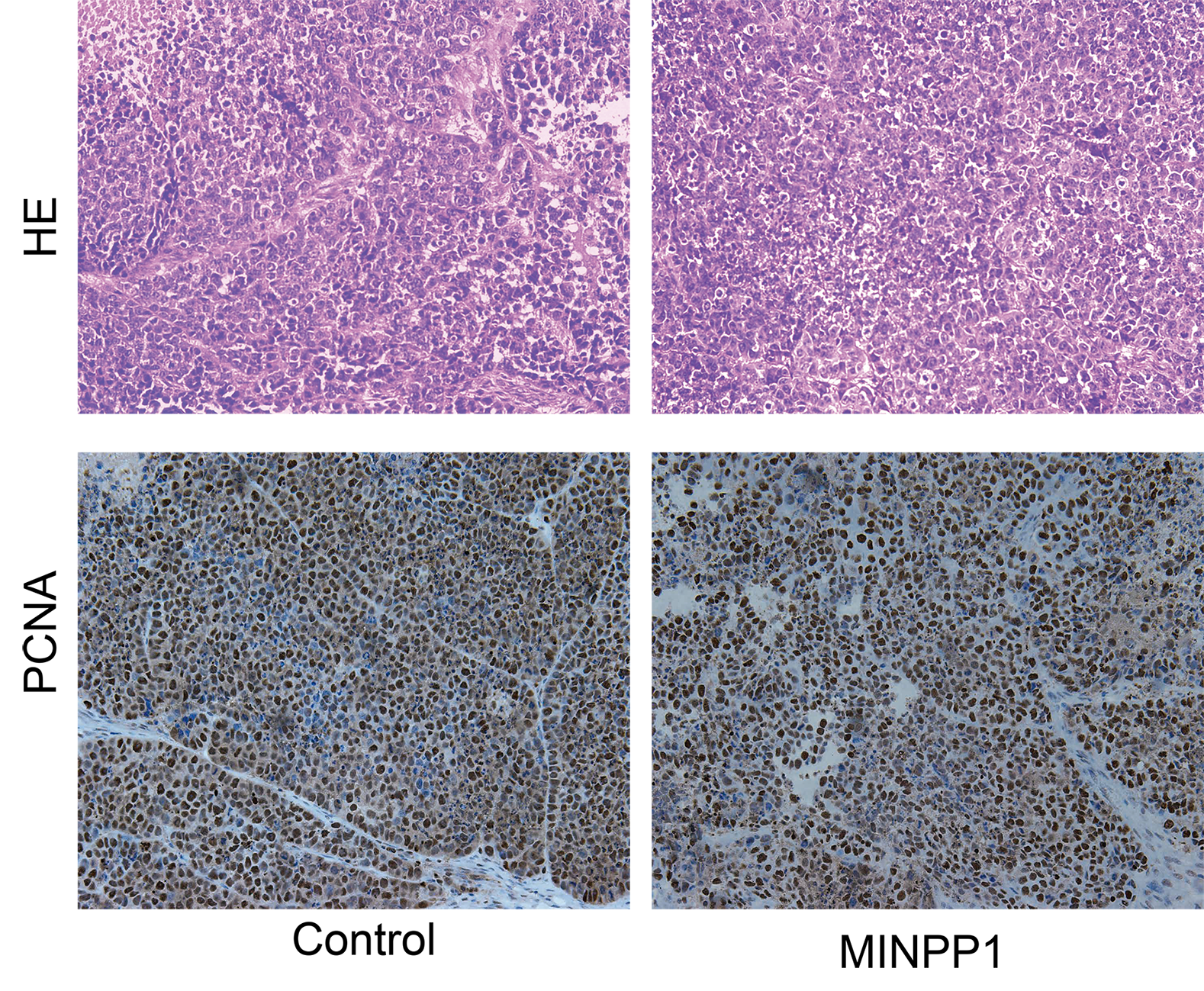

Supplement: Supplementary file 3 — Additional file 3: Figure S3. Results of HE staining and IHC analysis for PCNA in xenograft tumor tissues obtained from nude mice treated with Hep3B cells overexpressing MINPP1 and control. [file 13046_2020_1803_MOESM3_ESM.tif]

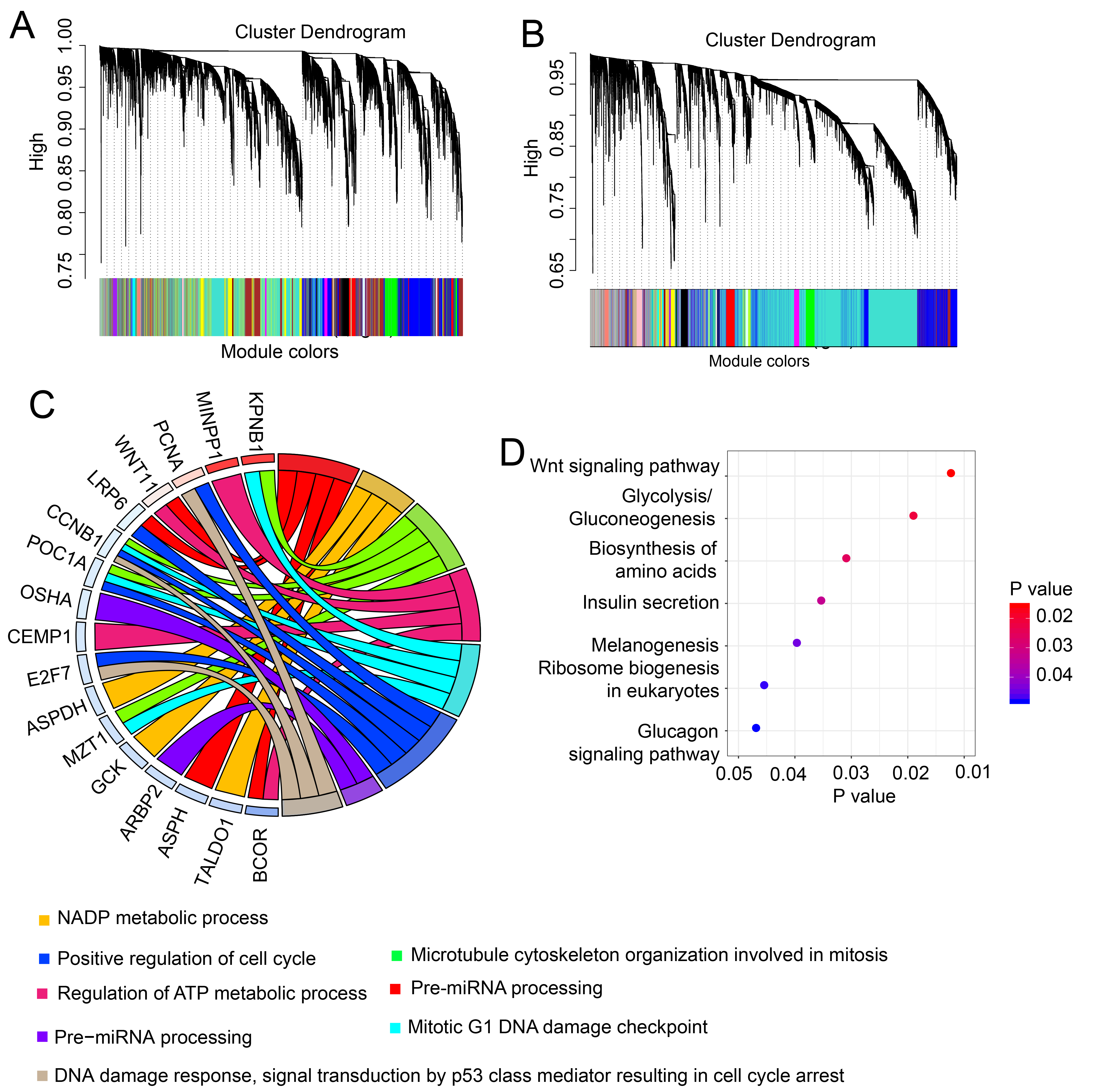

Supplement: Supplementary file 4 — Additional file 4: Figure S4. Validation of biological function of MINPP1 in the database. WGCNA algorithm was used to analyze the gene expression profiles related to MINPP1 in (A) GSE55092 and (B) TCGA cohorts. (C) GO analysis of the association between genes and biological processes. (D) KEGG analysis of genes involved in pathways. [file 13046_2020_1803_MOESM4_ESM.tif]

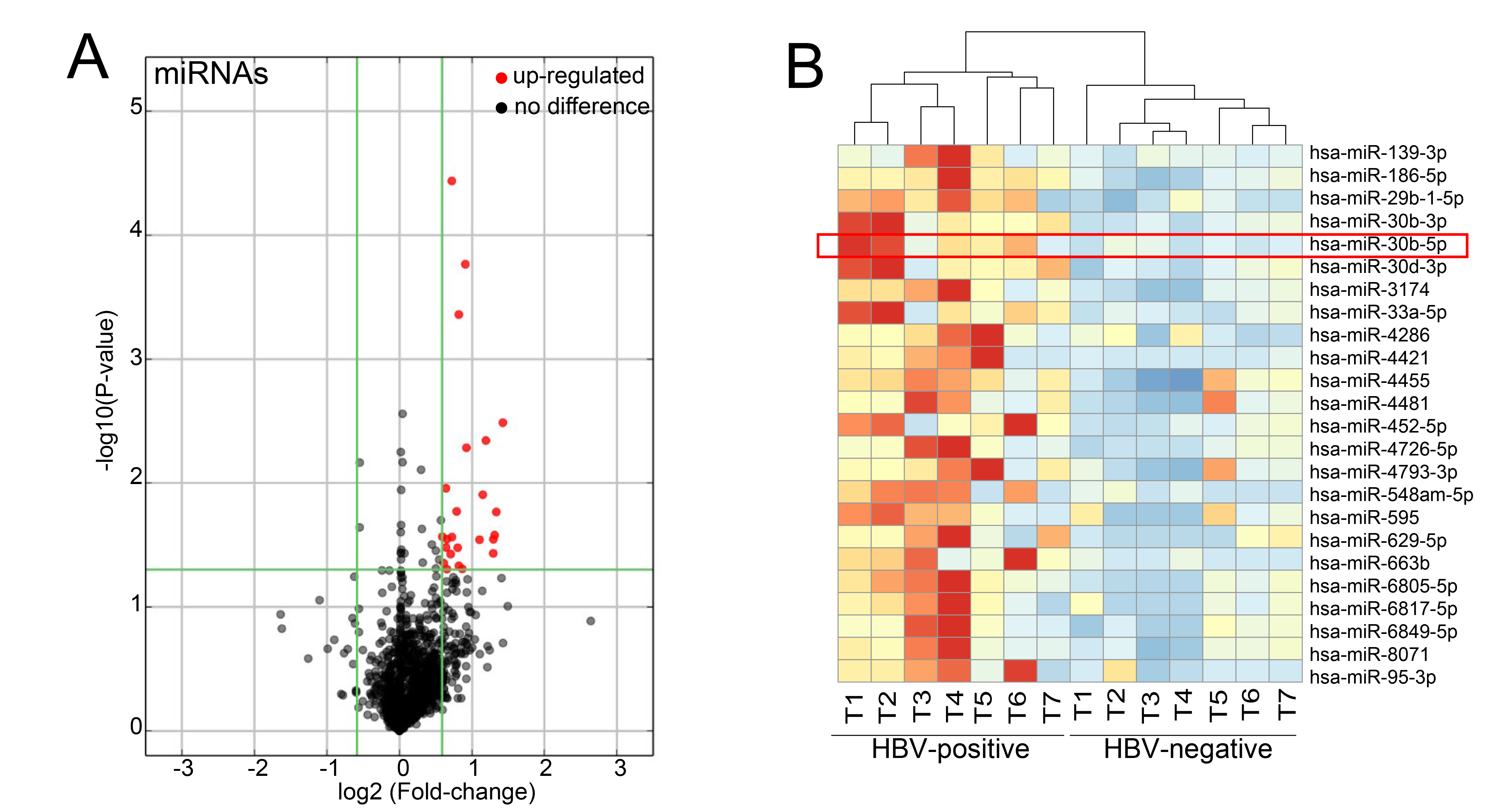

Supplement: Supplementary file 5 — Additional file 5: Figure S5. miRNAs differentially expressed between HBV-positive and HBV-negative HCC tissues. (A) Volcano Plot showing the differentially expressed miRNAs between HBV-positive and HBV-negative HCC tissues. (B) heatmap clustering of the up-regulated miRNAs in HBV-positive and HBV-negative tissues. [file 13046_2020_1803_MOESM5_ESM.tif]

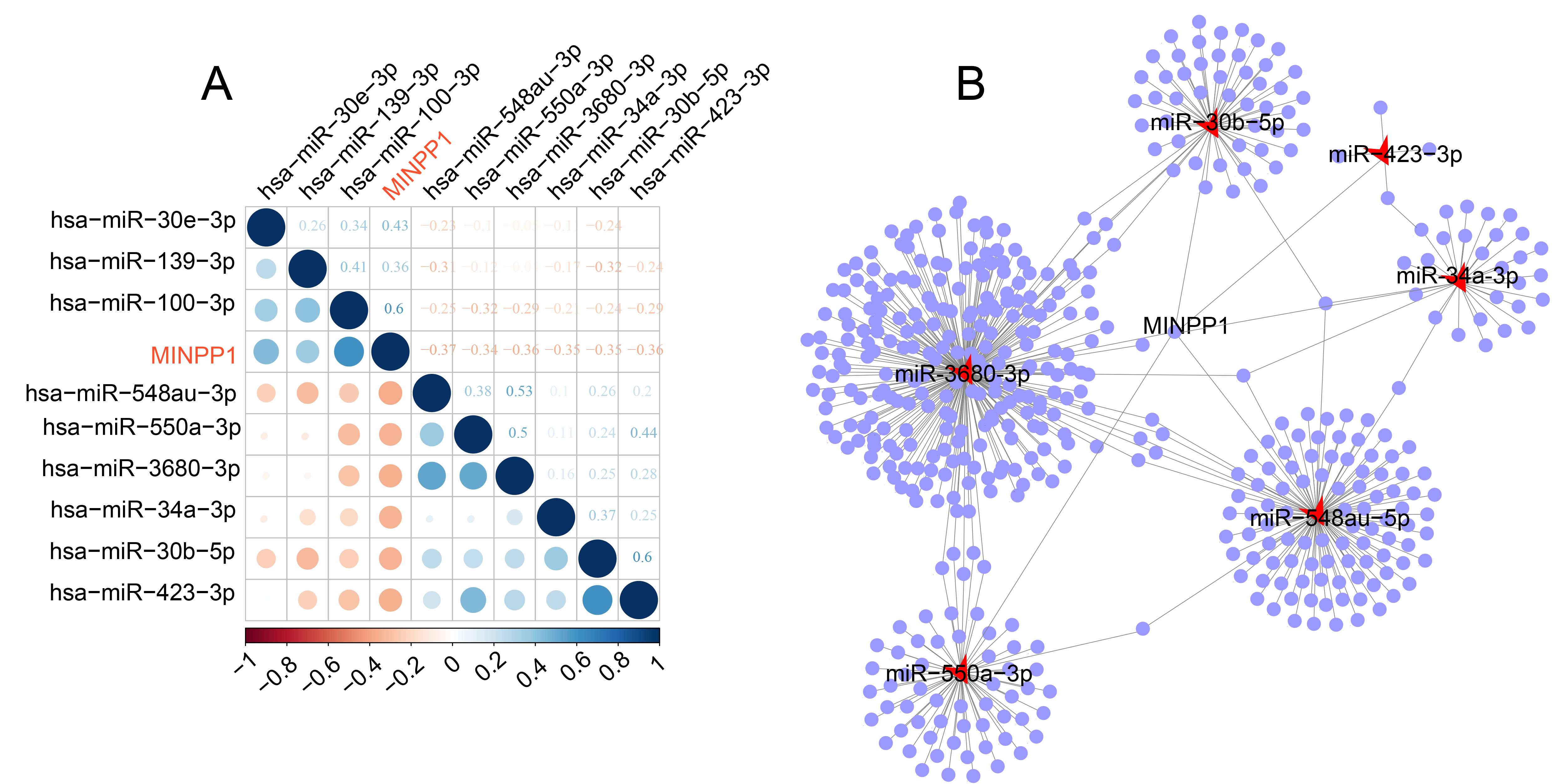

Supplement: Supplementary file 6 — Additional file 6: Figure S6. The association between MINPP1 and miRNA-30b-5p was validated in the database. (A) Correlation between MINPP1 and predicted miRNAs was analyzed in the TCGA cohort. (B) The interaction network of MINPP1 and the predicted miRNA. [file 13046_2020_1803_MOESM6_ESM.tif]

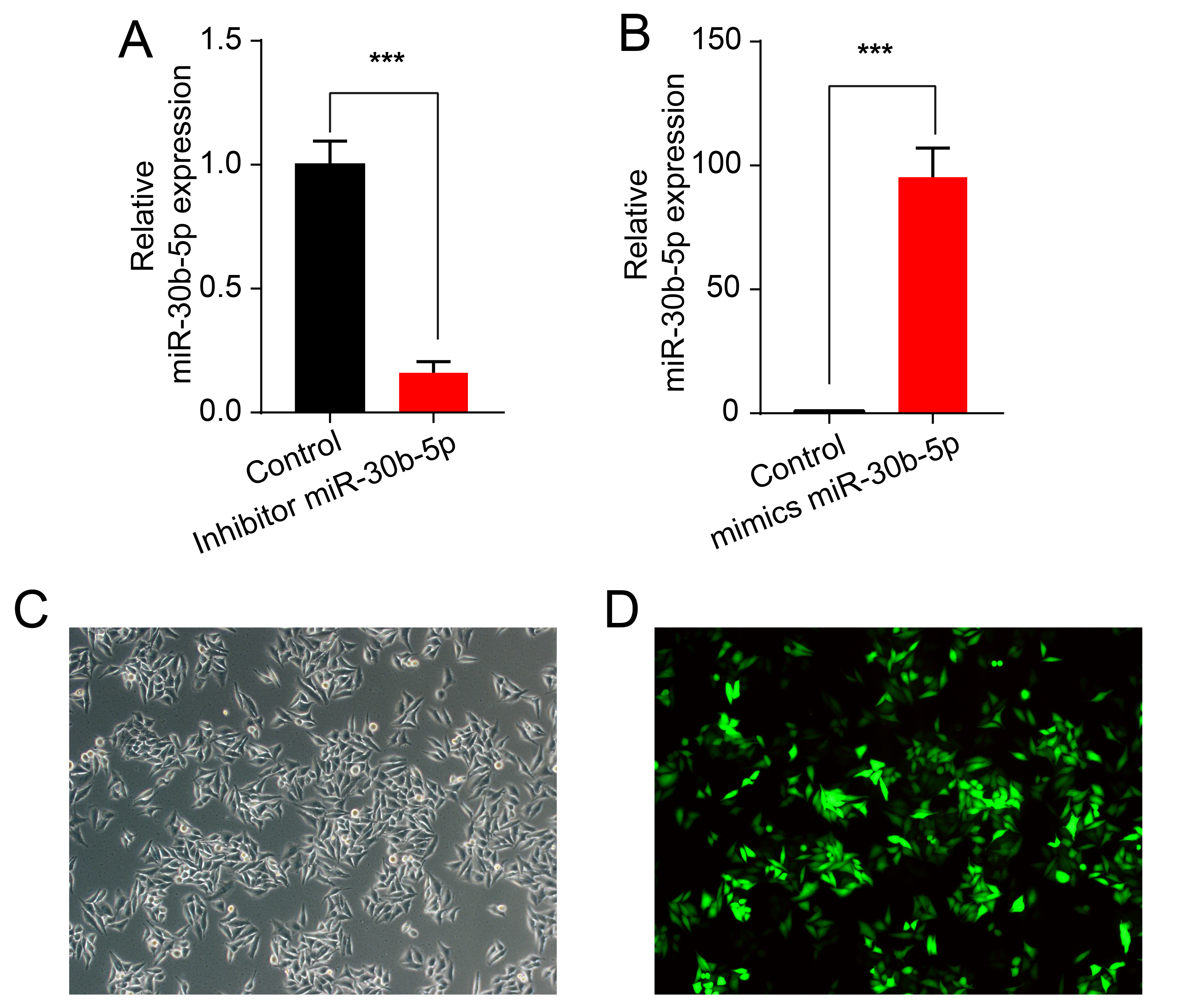

Supplement: Supplementary file 7 — Additional file 7: Figure S7. Measure on transfection efficiency of inhibitors and mimics of miRNA-30b-5p. The transfection efficiency of miRNA-30b-5p in Hep3B cells. The relative expression levels of miRNA-30b-5p after transfection with (A) inhibitors and (B) mimics in Hep3B cells. (C) The image of cell before transfected with inhibitors of miRNA-30b-5p. (D) The transfection efficiency of inhibitors of miRNA-30b-5p was measured by green fluorescence intensity from GFP. ***P < 0.001. [file 13046_2020_1803_MOESM7_ESM.tif]

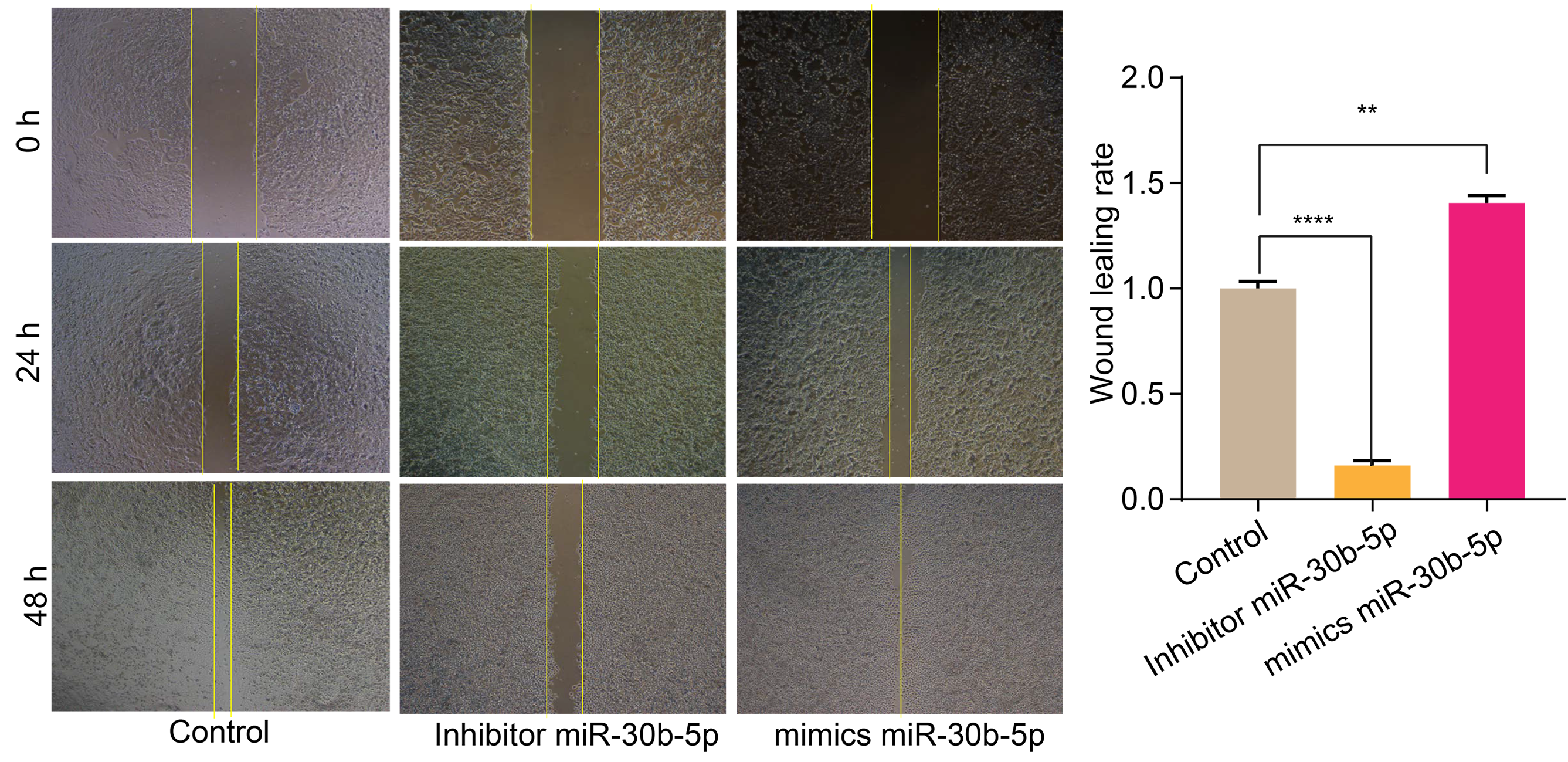

Supplement: Supplementary file 8 — Additional file 8: Figure S8. Scrape motility were measured by cell migration assays at 0, 24, and 48 h in the Hep3B cells with miRNA-30b-5p inhibition and overexpression. The wound healing rate was tested by Image J software. [file 13046_2020_1803_MOESM8_ESM.tif]

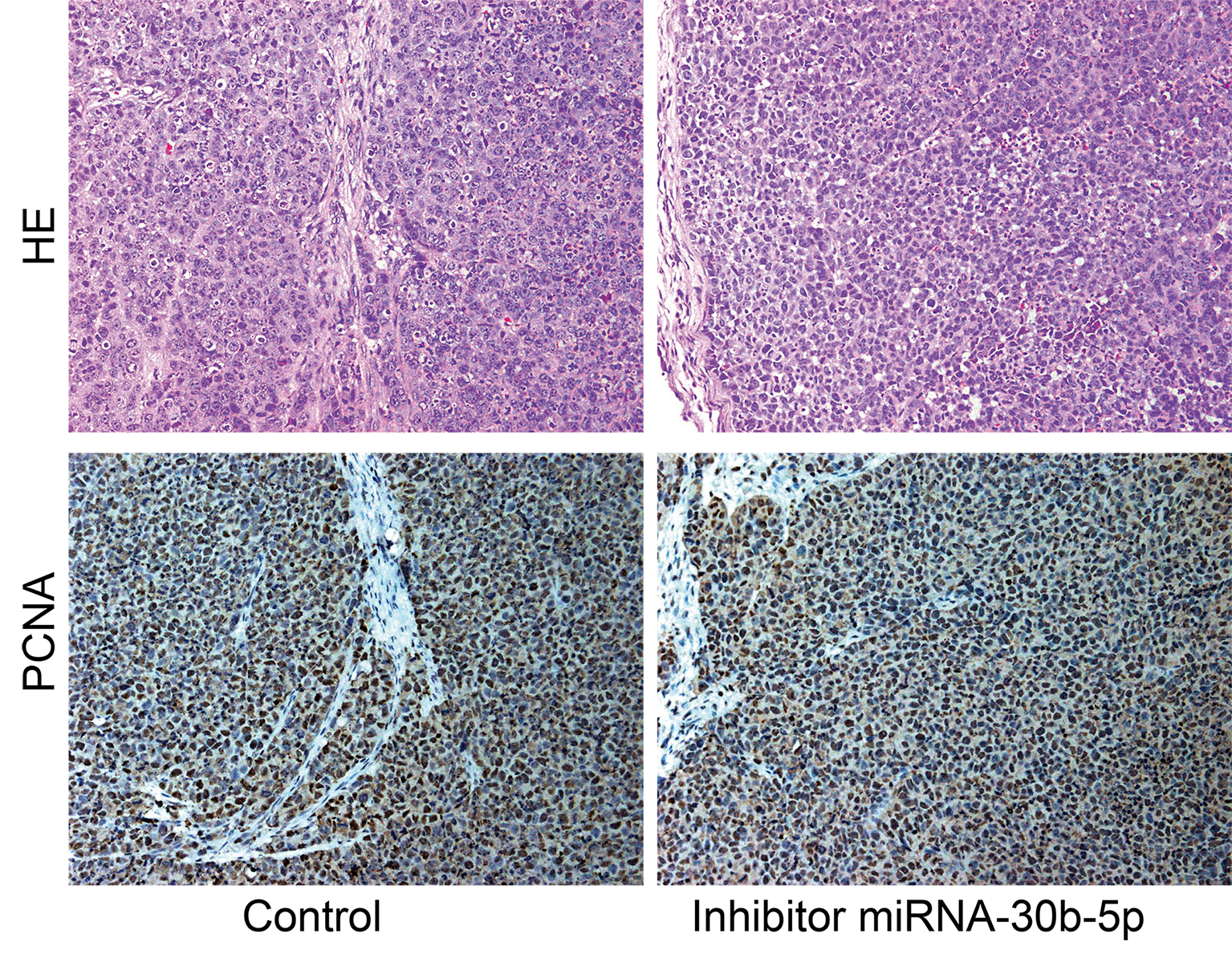

Supplement: Supplementary file 9 — Additional file 9: Figure S9. Results of HE staining and IHC analysis of PCNA in xenograft tumors from nude mice injected with Hep3B cells treated with the inhibitor of miRNA-30b-5p and control. [file 13046_2020_1803_MOESM9_ESM.tif]

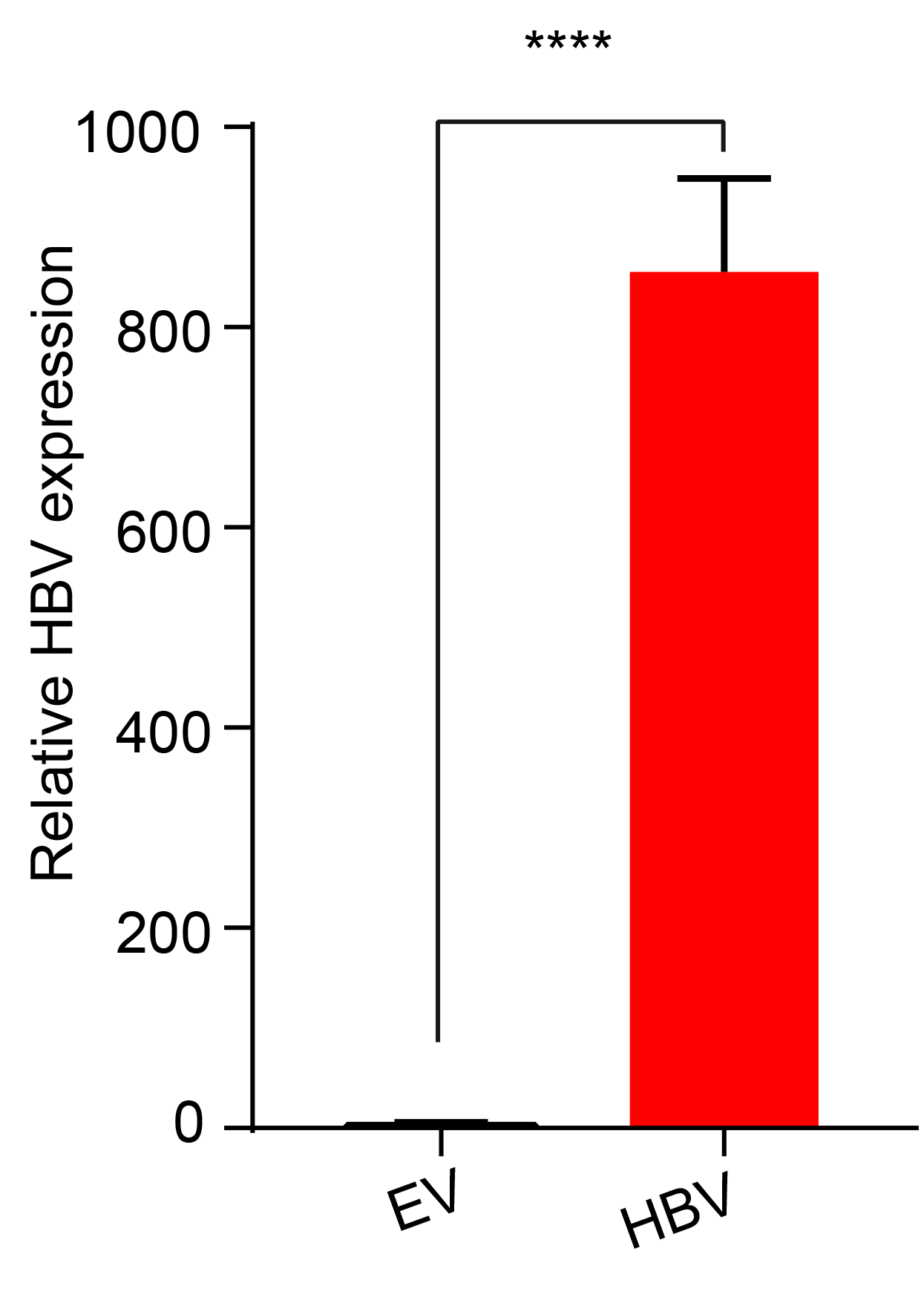

Supplement: Supplementary file 10 — Additional file 10: Figure S10. Relative expression levels of HBV after transfection with HBV DNA (pHBV1.3) into Huh7 cells. ****P < 0.0001. [file 13046_2020_1803_MOESM10_ESM.tif]

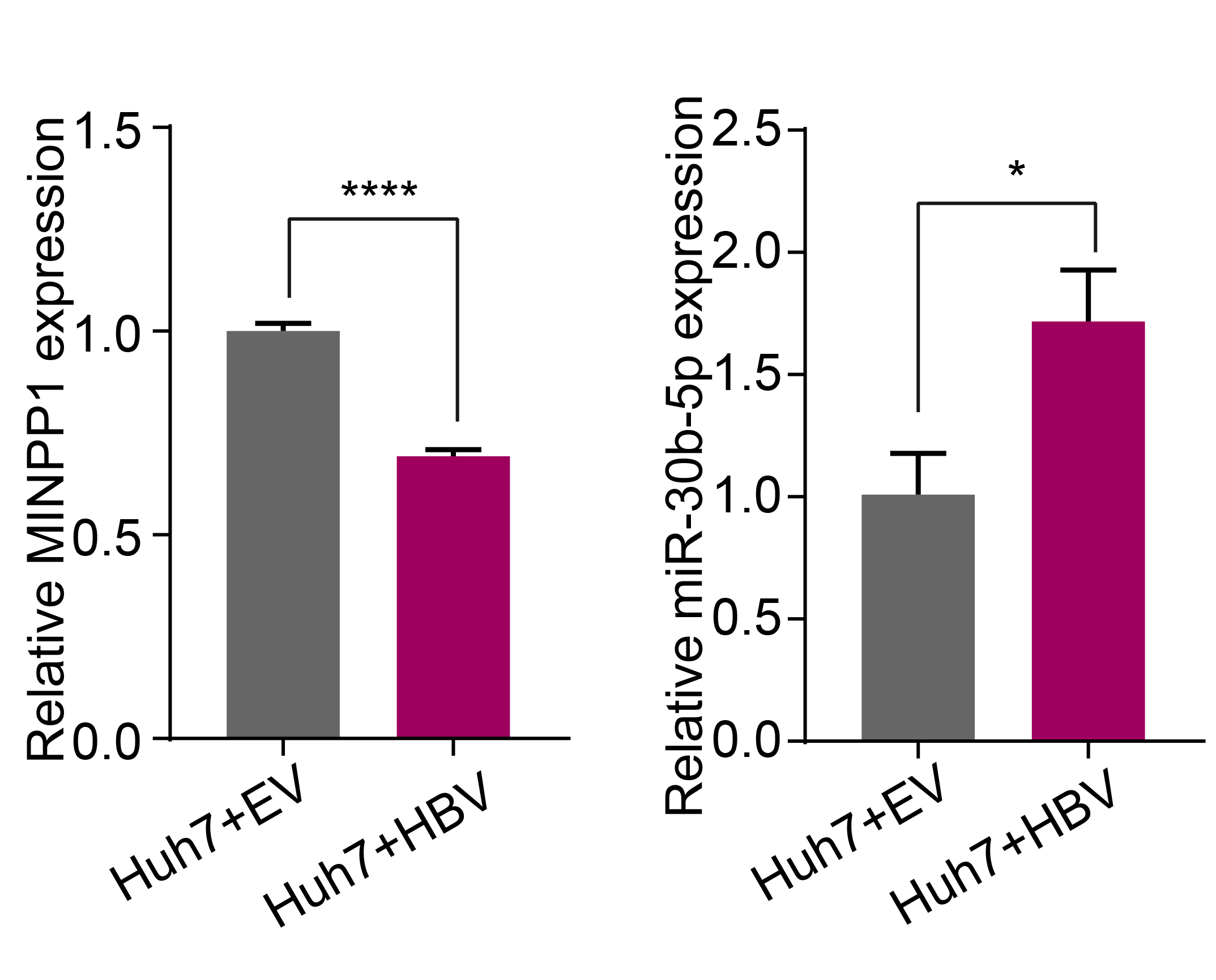

Supplement: Supplementary file 11 — Additional file 11: Figure S11. The expression level of MINPP1 (left) and miRNA-30b-5p (right) was examined by RT-qPCR after the Huh7 cells were transfected with pHBV1.3 or control. *P < 0.05, ****P < 0.0001. [file 13046_2020_1803_MOESM11_ESM.tif]

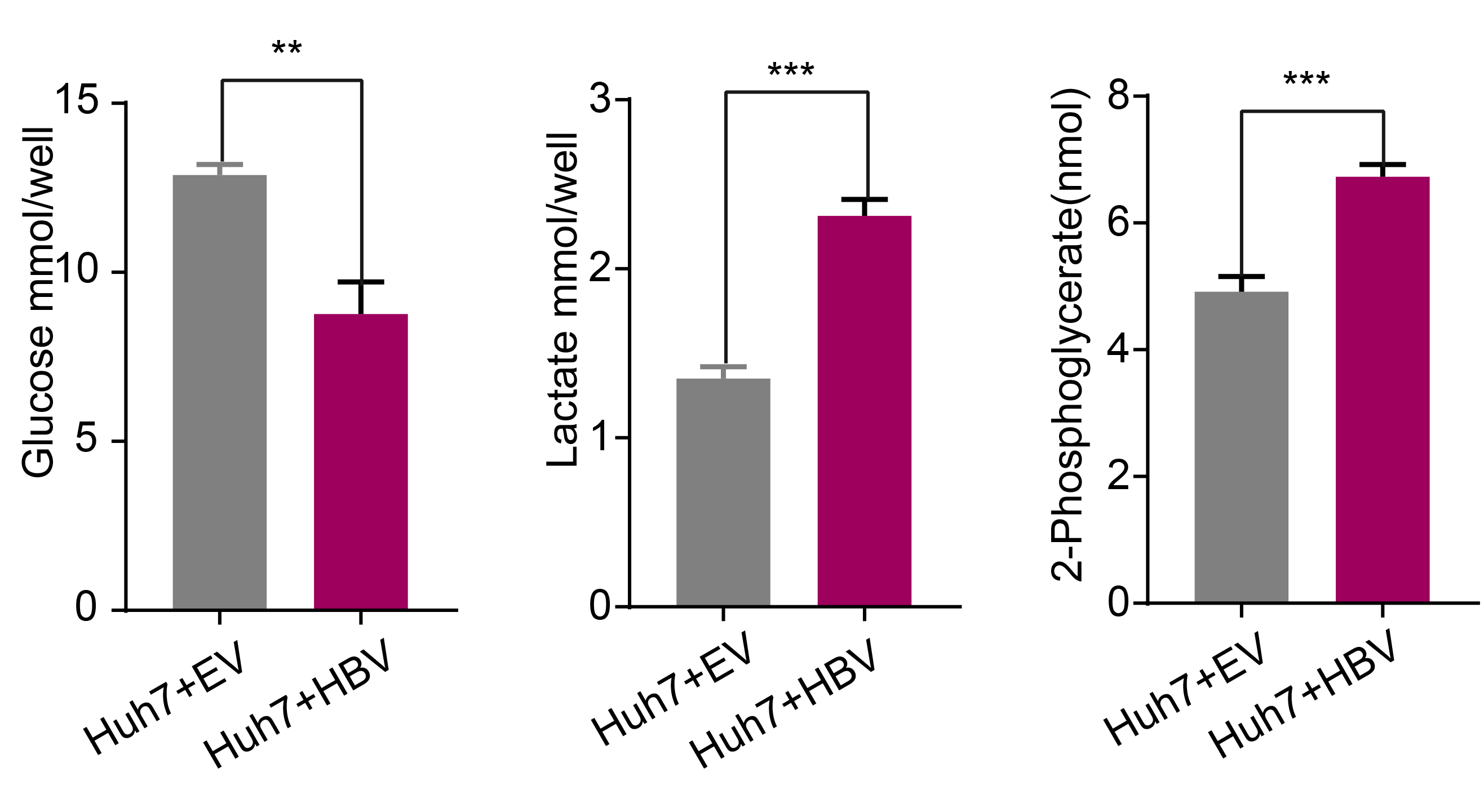

Supplement: Supplementary file 12 — Additional file 12: Figure S12. Cellular glucose (left), lactate (middle), and 2-PG (right) levels were measured in the Huh7 cells transfected with pHBV1.3 and control empty vector (EV). **P < 0.01, ***P < 0.001. [file 13046_2020_1803_MOESM12_ESM.tif]

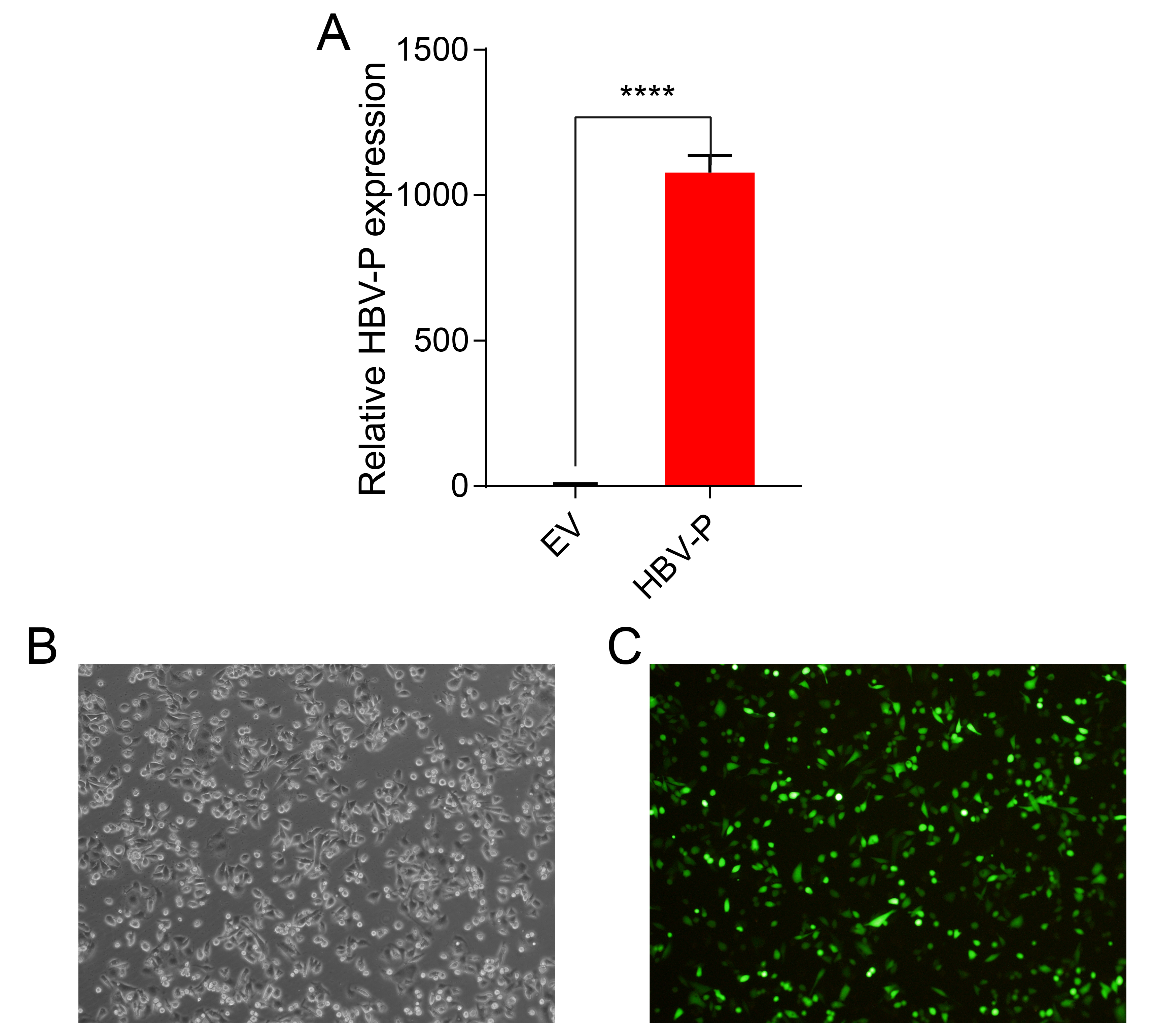

Supplement: Supplementary file 13 — Additional file 13: Figure S13. Measure on transfection efficiency of viral proteins HBp plasmids. (A) Relative expression levels of HBp after transfection with viral proteins HBp plasmids into Huh7 cells. (B) The image of cell before transfected with viral proteins HBp plasmids. (C) The transfection efficiency of viral proteins HBp plasmids was measured by green fluorescence intensity from GFP. ****P < 0.0001. [file 13046_2020_1803_MOESM13_ESM.tif]
