# Supplementary material for: Hepatitis B virus P protein initiates glycolytic bypass in HBV-related hepatocellular carcinoma via a FOXO3/miRNA-30b-5p/MINPP1 axis
Source: J Exp Clin Cancer Res. 2021 Jan 4;40:1. doi: 10.1186/s13046-020-01803-8 (PMC7779247; doi:10.1186/s13046-020-01803-8)
Supplement: Supplementary file 14 — Additional file 14: Table S1. The clinical characteristic of HBV-positive and HBV-negative HCC patient using in the microarray analysis. Table S2. The primers for RT-qPCR and sequences using in this study. Table S3. Different expression mRNAs between HBV-positive and HBV-negative HCC samples. Table S4. Different expression miRNAs between HBV-positive and HBV-negative HCC samples. [file 13046_2020_1803_MOESM14_ESM.docx]

Table S1. The clinical characteristic of HBV-positive and HBV-negative HCC patient using in the microarray analysis.

| **HCC Sample** | **Age (year)** | **Gender** | **HBSAg** | **HBeAg** | **HBcAb** | **HAV-IgM, HCV-IgG, HEV-IgG** | **HBV-DNA (IU/ml)** | **AFP (ng/ml)** | **ALP (U/l)** | **Total bilirubin (umol/l)** | **Ascites (medical image)** | **Tumor Size (cm)** | **Tumor metastasis** | **Pathological**  **type** | **Pathological**  **stage** |  |
| --- | --- | --- | --- | --- | --- | --- | --- | --- | --- | --- | --- | --- | --- | --- | --- | --- |
| HBV(+) 1 | 44 | man | Positive | Positive | Positive | Negative | 6.7x10^4^ | 482 | 60 | 27.3 | Negative | 6.5x5.5 | bile duct | Hepatocellular carcinoma | III-IV |  |
| HBV(+) 2 | 54 | man | Positive | Positive | Positive | Negative | 9.5x10^5^ | 46 | 71 | 35 | Positive | 5.6x3.7 | None | Hepatocellular carcinoma | II |  |
| HBV(+) 3 | 55 | man | Positive | Positive | Positive | Negative | 1.84x10^5^ | 439 | 84 | 26.66 | Positive | 7x5.5 | None | Hepatocellular carcinoma | II |  |
| HBV(+) 4 | 52 | man | Positive | Positive | Positive | Negative | 1.41x10^6^ | 83 | 109 | 43.7 | Positive | 4x4 | Gastric area | Hepatocellular carcinoma | IV |  |
| HBV(+) 5 | 46 | man | Positive | Positive | Positive | Negative | 1.63x10^4^ | 345 | 10 | 11.3 | Negative | 2.3x2.3 | Portal lymph  node | Hepatocellular carcinoma | IV |  |
| HBV(+) 6 | 66 | man | Positive | Positive | Positive | Negative | 5.4x10^5^ | 123.5 | 132.9 | 18.3 | Negative | 5.6x9 | Lung | Hepatocellular carcinoma | III-IV |  |
| HBV(+) 7 | 56 | man | Positive | Positive | Positive | Negative | 3.5x10^4^ | 484.6 | 22 | 24.7 | Positive | 4.7x5 | None | Hepatocellular carcinoma | III |  |
| HBV(-) 1 | 52 | man | — | — | — | Negative | — | 12 | 54.2 | 28.3 | Negative | 4.3x5 | bile duct | Hepatocellular carcinoma | III |  |
| HBV(-) 2 | 42 | man | — | — | — | Negative | — | 168.4 | 15 | 41 | Positive | 8x6 | None | Hepatocellular carcinoma | III-IV |  |
| HBV(-) 3 | 60 | man | — | — | — | Negative | — | 45.2 | 47.4 | 37.6 | Negative | 5x4.5 | Gastric area | Hepatocellular carcinoma | II |  |
| HBV(-) 4 | 73 | man | — | — | — | Negative | — | 23.5 | 54 | 115.6 | Positive | 4.5x3.5 | None | Hepatocellular carcinoma | II |  |
| HBV(-) 5 | 70 | woman | — | — | — | Negative | — | 6 | 146 | 398.6 | Positive | 3.5x2.5 | None | Hepatocellular carcinoma | II-III |  |
| HBV(-) 6 | 56 | man | — | — | — | Negative | — | 12.6 | 31 | 29 | Negative | 4.2x3.5 | Portal lymph  node | Hepatocellular carcinoma | I-II |  |
| HBV(-) 7 | 48 | woman | — | — | — | Negative | — | 1841 | 264 | 43.7 | Positive | 15.5x8 | bile duct | Hepatocellular carcinoma | II |  |
|  |  |  |  |  |  |  |  |  |  |  |  |  |  |  |  |  |

Table S2 The primers for RT-qPCR and sequences using in this study.

| **siRNA or inhibitor sequecne** | **sense（5'-3'）** | **antisense（5'-3'）** |
| --- | --- | --- |
| miRNA-30b-5p inhibitor | AGCUGAGUGUAGGAUGUUUACA |  |
| miRNA-30b-5p mimics | UGUAAACAUCCUACACUCAGCU | AGCUGAGUGUAGGAUGUUUACA |
| si-FOXO3-homo-1094 | GCUGUCUCCAUGGACAAUATT | UAUUGUCCAUGGAGACAGCTT |
| si-MINPP1-homo-896 | CCUGGCAAUUAAAGGUGUUTT | AACACCUUUAAUUGCCAGGTT |
| si-MINPP1-homo-1071 | GCAGUUGAACAGAAACAAATT | UUUGUUUCUGUUCAACUGCTT |
| si-MINPP1-homo-1258 | CCUCGAACCUGAUAUUUGUTT | ACAAAUAUCAGGUUCGAGGTT |
| Negtive control | UUCUCCGAACGUGUCACGUTT | ACGUGACACGUUCGGAGAATT |
| **FISH probe sequence** |  |  |
| MINPP1 FISH probe | TGA+AACAAGGTGCAGCTGGA+TC |  |
| miRNA-30b-5p FISH probe | AGCTG+AGTGT+AGGATGTT+TACA |  |
| Negative control FISH probe | TGCTTTGCACGGTAACGCCTGTTTT |  |
| 18S FISH probe | CTGCCTTCCTTGGATGTGGTAGCCGTTTC |  |
| **Primer sequence** | **Forward（5'-3'）** | **Reverse（5'-3'）** |
| MINPP1 | GTCGCTCAGCCCCTATTTCG | TGCGGATCTGTTTGACCGTG |
| GAPDH | GGAGCGAGATCCCTCCAAAAT | GGCTGTTGTCATACTTCTCATGG |
| FOXO3 | CGGACAAACGGCTCACTCT | GGACCCGCATGAATCGACTAT |
| miRNA-30b-5p | Designed and purchased from Ribobio (Guangzhou, China) | |
| U6 | Designed and purchased from Ribobio (Guangzhou, China) | |

Table S3. Different expression mRNAs between HBV-positive and HBV-negative HCC samples.

| **Gene symbol** | **Fold change** | **P-value** | **Regulation** |
| --- | --- | --- | --- |
| UGT3A2 | 2.3229999 | 0.037708875 | up |
| PRSS57 | 2.5090883 | 0.007643876 | up |
| TP53TG3D | 3.1748939 | 0.021716547 | up |
| FSTL4 | 7.9900213 | 0.002037109 | up |
| MTBP | 2.681648 | 0.033295494 | up |
| SIPA1L2 | 2.3483695 | 0.027678199 | up |
| AX748369 | 2.1540426 | 0.019918359 | up |
| KISS1R | 3.9514037 | 0.024647 | up |
| DISP1 | 2.0526826 | 0.025426102 | up |
| STMN1 | 2.0507745 | 0.047667379 | up |
| DNAH1 | 2.0168428 | 0.04689983 | up |
| CAPZA3 | 5.3272031 | 0.033037847 | up |
| RASGEF1B | 2.0207507 | 0.041002761 | up |
| OR2Z1 | 2.020098 | 0.017238656 | up |
| NTNG1 | 2.0290677 | 0.043297717 | up |
| MLPH | 5.9877223 | 0.011524214 | up |
| ANKRD46 | 2.1163828 | 0.035943425 | up |
| RAI1 | 2.956685 | 0.042548271 | up |
| TBC1D31 | 4.1663939 | 0.004220211 | up |
| WDR67 | 2.2101644 | 0.021007765 | up |
| C8orf76 | 2.0526809 | 0.014752094 | up |
| IFT172 | 2.1529264 | 0.036922459 | up |
| LCE2D | 2.7844172 | 0.023521072 | up |
| SKIDA1 | 3.0717854 | 0.018203829 | up |
| KLHL34 | 5.6223033 | 0.028592801 | up |
| TPPP | 2.7326755 | 0.027978268 | up |
| DDI2 | 2.2106952 | 0.006821149 | up |
| FLJ30838 | 2.1546802 | 0.026853551 | up |
| HTR4 | 3.1872805 | 0.039596409 | up |
| DPYD | 2.0662357 | 0.00053628 | up |
| HSP90AB4P | 2.3709898 | 0.047334064 | up |
| AC026740.1 | 2.4434126 | 0.025489783 | up |
| RHOXF1 | 2.2900386 | 0.013232055 | up |
| SYN1 | 2.8197085 | 0.021356107 | up |
| RHCE | 2.9374114 | 0.043374455 | up |
| PROS1 | 2.0136121 | 0.018331451 | down |
| ZBTB5 | 2.2817913 | 0.014779853 | down |
| AADAT | 4.8326946 | 0.0053382 | down |
| KDELC2 | 2.1459654 | 0.041277095 | down |
| LMAN1 | 2.2474876 | 0.003510664 | down |
| EI24 | 2.785733 | 0.013447972 | down |
| P4HTM | 2.058525 | 0.048173148 | down |
| C2orf88 | 2.3986233 | 0.023133876 | down |
| ATOH8 | 2.3222079 | 0.03445712 | down |
| MPZL3 | 4.4498176 | 0.000523914 | down |
| ADRA2C | 2.6338393 | 0.010421059 | down |
| PCDHGA2 | 3.9955224 | 0.045865182 | down |
| UBAC2 | 2.177636 | 0.027454778 | down |
| PPEF1 | 3.2891033 | 0.028178677 | down |
| EXOC5 | 2.0548096 | 0.019043522 | down |
| GPR75 | 2.3218256 | 0.003787448 | down |
| ISPD | 2.049955 | 0.021010827 | down |
| IGFL2 | 2.9404338 | 0.037440146 | down |
| DBP | 2.7597604 | 0.001061441 | down |
| APOL3 | 2.380017 | 0.03910842 | down |
| EXPH5 | 3.9457307 | 0.035195022 | down |
| MPZL2 | 2.2939861 | 0.016152957 | down |
| ZZEF1 | 2.2743631 | 0.029213693 | down |
| TSC22D1 | 2.689988 | 0.022296197 | down |
| DIRC2 | 2.0463068 | 0.038861805 | down |
| TTC30A | 2.1185116 | 0.031092824 | down |
| ACAP2 | 2.0816265 | 0.025246532 | down |
| NHLRC2 | 2.0086799 | 0.001283637 | down |
| ROBO2 | 4.0391021 | 0.044363095 | down |
| FAM118B | 2.2201816 | 0.030130784 | down |
| ENPP4 | 2.4603382 | 0.00256278 | down |
| PGM2 | 3.2484339 | 0.016674622 | down |
| HIATL1 | 2.3125637 | 0.015126412 | down |
| ENTPD7 | 2.1392602 | 0.028131059 | down |
| PARP16 | 2.007941 | 0.021494739 | down |
| PCDHGA5 | 2.2709795 | 0.041536939 | down |
| IGFBP5 | 2.5861101 | 0.001439548 | down |
| RAP2C | 2.2211254 | 0.008652676 | down |
| DNAJB1 | 2.7155402 | 0.035390185 | down |
| HEPN1 | 5.3502184 | 0.032260902 | down |
| PIK3R1 | 2.1755472 | 0.039873388 | down |
| TMEM9B | 2.0100781 | 0.010165898 | down |
| SGPL1 | 2.3793268 | 0.001648601 | down |
| STK32C | 2.5191003 | 0.029310481 | down |
| C4orf32 | 2.3734594 | 0.010757335 | down |
| DNMBP | 2.3603909 | 0.005407183 | down |
| LRFN3 | 2.069729 | 0.049810858 | down |
| SYPL1 | 2.0849586 | 0.008528688 | down |
| GALNT2 | 2.025207 | 0.034459214 | down |
| MFAP3L | 3.0197142 | 0.018674801 | down |
| EMC10 | 2.9382612 | 0.001623173 | down |
| ZRANB1 | 2.0628215 | 0.005874473 | down |
| GLUD1 | 2.8042434 | 0.028903124 | down |
| MINPP1 | 2.2269731 | 0.002304837 | down |
| LDHC | 3.7048597 | 0.027166277 | down |
| SIX1 | 3.9887377 | 0.047409209 | down |
| APOL2 | 2.1554787 | 0.024983885 | down |
| CADM4 | 3.0685452 | 0.044153042 | down |
| SATB2 | 3.0806675 | 0.011306037 | down |
| FAM92A1 | 4.8629259 | 0.035483311 | down |
| CST1 | 3.5966898 | 0.034148818 | down |
| TGFBI | 2.0990347 | 0.009861286 | down |
| TBC1D1 | 2.1324532 | 0.02616591 | down |
| MEPCE | 2.0966679 | 0.034399785 | down |
| PDZD8 | 2.6903084 | 0.011862069 | down |
| TMEM248 | 2.5534134 | 0.006897915 | down |
| RNF157 | 2.7939075 | 0.021505888 | down |
| TMEM182 | 2.6517295 | 0.029085755 | down |
| HS3ST1 | 2.1674549 | 0.016316984 | down |
| SMPD1 | 2.1228163 | 0.026820949 | down |
| ERLIN1 | 2.0921932 | 0.0020615 | down |
| POLR1E | 2.0020694 | 0.021793799 | down |
| CDCP1 | 2.1583634 | 0.027878307 | down |
| WNT5A | 3.0924094 | 0.020712286 | down |
| UBP1 | 2.4386514 | 0.00038499 | down |

Table S4. Different expression miRNAs between HBV-positive and HBV-negative HCC samples..

| **miRNAs** | **Fold Change** | **P-value** | **Regulation** |
| --- | --- | --- | --- |
| hsa-miR-139-3p | 1.5066755 | 0.027104501 | up |
| hsa-miR-186-5p | 1.8781899 | 0.00017153 | up |
| hsa-miR-29b-1-5p | 2.5255515 | 0.017099704 | up |
| hsa-miR-30b-3p | 2.2182224 | 0.012413229 | up |
| hsa-miR-30b-5p | 1.8975625 | 0.005188425 | up |
| hsa-miR-30d-3p | 2.4558944 | 0.028418581 | up |
| hsa-miR-3174 | 2.2851299 | 0.004539188 | up |
| hsa-miR-33a-5p | 2.1508594 | 0.028657106 | up |
| hsa-miR-4286 | 1.8241256 | 0.049271485 | up |
| hsa-miR-4421 | 1.6529667 | 0.027282997 | up |
| hsa-miR-4455 | 1.5759754 | 0.028360574 | up |
| hsa-miR-4481 | 1.5752345 | 0.049522459 | up |
| hsa-miR-452-5p | 2.4523787 | 0.036913836 | up |
| hsa-miR-4726-5p | 1.5608146 | 0.011024441 | up |
| hsa-miR-4793-3p | 1.7287592 | 0.016926285 | up |
| hsa-miR-548am-5p | 1.5278853 | 0.044229037 | up |
| hsa-miR-595 | 1.7457272 | 0.033233015 | up |
| hsa-miR-629-5p | 1.631878 | 0.037468056 | up |
| hsa-miR-663b | 2.6863513 | 0.003257151 | up |
| hsa-miR-6805-5p | 1.7638026 | 0.000436938 | up |
| hsa-miR-6817-5p | 1.650185 | 3.65053E-05 | up |
| hsa-miR-6849-5p | 1.7655251 | 0.046612212 | up |
| hsa-miR-8071 | 1.5612874 | 0.033059006 | up |
| hsa-miR-95-3p | 2.4839777 | 0.026309891 | up |
